# Down-Regulation of Oncogene *c-myb* Specifically by Carbazole Derivative Through Opposing Effects on Different Quadruplex Structures of Gene Promoter for Cancer Treatment

**DOI:** 10.3390/ijms26178299

**Published:** 2025-08-27

**Authors:** Siyi Wang, Jihai Liang, Jiahui Zhang, Dongsheng Ji, Zhi-Shu Huang, Ding Li

**Affiliations:** School of Pharmaceutical Sciences, Sun Yat-sen University, Guangzhou University City, Guangzhou 510006, China; wangsy86@mail2.sysu.edu.cn (S.W.); liangjh225@mail2.sysu.edu.cn (J.L.); zhangjh265@mail2.sysu.edu.cn (J.Z.); jidsh@mail2.sysu.edu.cn (D.J.); ceshzs@mail.sysu.edu.cn (Z.-S.H.)

**Keywords:** *c-myb*, carbazole derivative, i-motif, G-quadruplex, transcriptional regulation, anti-tumor

## Abstract

Cancer is one of the leading causes of human mortality worldwide, and aberrant expression of the *c-myb* oncogene is closely associated with the development of numerous malignancies. The *c-myb* promoter region contains G-rich and C-rich sequences capable of forming G-quadruplex (G4) and i-motif (IM or C-quadruplex) structures, respectively. These secondary structures function as “molecular switches” for gene transcriptional regulation and represent promising targets for novel anti-tumor therapeutics. Through extensive screening, we identified carbazole derivative **G51** as a unique dual-targeting ligand that simultaneously destabilized the i-motif and stabilized the G-quadruplex, consequently suppressing *c-myb* expression efficiently. In comparison, the single-targeting ligand **G50**, which could specifically bind to and unfold the G-quadruplex only, exhibited significantly weaker anti-tumor activity than **G51**. Notably, **G51** showed potent anti-tumor efficacy in a human colorectal cancer xenograft model without significant toxicity to vital organs. **G51**, as a dual-targeting ligand, had specific binding to *c-myb* promoter quadruplexes, with destabilization of the i-motif and concurrent stabilization of the G-quadruplex. This opposing effect could provide a good opportunity for specific gene regulation, with great potential for further development of a precise therapeutic agent. This study provides a novel example for a practical therapeutic approach through coordinated gene quadruplex modulations, which sets up a good foundation for developing high-efficacy anti-tumor drugs without significant side effects.

## 1. Introduction

Cancer is a leading cause of mortality worldwide, with both incidence and death rates increasing each year. According to the World Health Organization (WHO), approximately 20 million new cancer cases and 10 million cancer-related deaths occurred globally in 2020. These numbers are projected to rise by 1.5-fold by 2040 [1]. The development of cancer is influenced by various factors, among which the abnormal activation of proto-oncogenes plays a pivotal role [2]. The *c-myb* proto-oncogene was originally identified through reverse transcription of retroviral sequences from AMV and E26, with its encoded C-MYB protein regulating essential cellular processes, such as differentiation, proliferation, and maintenance of homeostasis [3]. Aberrant expression of *c-myb* is closely associated with several malignancies, such as acute myeloid leukemia, breast cancer, and colorectal cancer [4,5]. The C-MYB protein exerts its oncogenic effects by modulating cell cycle progression, differentiation, apoptosis, and metastasis through interactions with transcription factors, cell cycle regulators, microRNAs (miRNAs), and non-coding RNAs (ncRNAs) [6,7,8,9].

Targeting *c-myb* gene expression with small molecules has emerged as a promising anti-tumor strategy. Some small molecules inhibiting the C-MYB protein have been reported and showed therapeutic potential [10,11]. In addition, anti-sense oligonucleotides (ASOs) targeting *c-myb* have been introduced into clinical trials for hematological malignancies [12]. Therefore, direct modulation of *c-myb* transcription offers an alternative approach for effective cancer therapy. Conventional cancer treatments, such as surgery, radiotherapy, and chemotherapy, have shown some therapeutic effects; however, these effects are limited due to incomplete tumor eradication, systemic toxicity, and poor specificity [13]. The importance of gene-level regulation in cancer therapy has attracted significant attention; in particular, non-canonical DNA secondary structures, including G-quadruplexes (G4s) and i-motifs (also named as IMs or C-quadruplexes) on gene promoters, play crucial roles in transcriptional control [14]. Under near-physiological conditions, various G-rich DNA oligomers can form G-quadruplexes, while complementary C-rich oligomers can form i-motif structures, especially under mild acidic or molecular crowding conditions [15]. It should be noted that these two types of structures appear differently within the cell cycle, and G-quadruplexes are enriched in the S phase, while i-motifs are prominent in the G1 phase [16]. These quadruplex structures can act as “molecular switches” regulating gene transcription via conformational transitions. G-quadruplexes are widely recognized as transcriptional repressors, and some ligands targeting G4 structures, such as CX-3543, CX-5461, and APTO-253, have been introduced into clinical trials [17]. In contrast, small molecules targeting i-motif structures have received much less attention, with their biological roles remaining unclear and controversial. For example, acridone derivative **B19** can stabilize *c-myc* promoter i-motif and down-regulate its gene transcription [18], while acridone derivative **B14** can stabilize *BCL-2* promoter i-motif and up-regulate its gene transcription [19]. To date, no i-motif-targeting compound has progressed to clinical trials.

*c-myb* gene promoter contains both G-rich and C-rich sequences, which can form G-quadruplex (G4) and i-motif structures, respectively [20,21]. Dual-targeting ligands that simultaneously interact with both G4 (primarily in S phase) and i-motif (primarily in G1 phase) can offer great potential for regulating gene expression very effectively across multiple stages of the cell cycle. Our previous research has identified bisacridine derivative **A06** as a dual-targeting ligand capable of disrupting both G4 and i-motif structures on the promoter of tumor suppressor gene retinoblastoma (RB), consequently increasing RB expression significantly and inhibiting tumor proliferation and metastasis [22]. This dual-modulation approach has allowed for reduced dosage of drug administration with decreased toxicity, resulting in an increased anti-tumor therapeutic index. Encouraged by these findings, we further screened our compound libraries for dual-targeting other oncogene promoter quadruplex structures and identified carbazole derivative **G51** as a promising candidate for the *c-myb* gene promoter. While **A06** disrupts both G-quadruplex and i-motif structures of the RB promoter, in the present study, it was found that **G51** showed opposing effects on different quadruplex structures, which could stabilize G-quadruplex while disassembling i-motif on the *c-myb* gene promoter. In comparison, a structurally similar compound **G50** specifically disrupted G-quadruplex without significant interaction with i-motif on the *c-myb* gene promoter. Our molecular- and cellular-level experiments showed that **G51** significantly down-regulated *c-myb* gene transcription and translation with very strong anti-tumor activity in comparison to **G50**. Furthermore, using a colorectal cancer xenograft model, our in vivo experiment showed that **G51** had potent anti-tumor activity without significant toxicity to major organs. These results increased our understanding of *c-myb* promoter quadruplex structures and their potential as therapeutic targets. This study also provided a promising approach for the practical development of highly specific anti-cancer agents targeting quadruplex structures of oncogene promoters.

## 2. Results

The *c-myb* promoter region contains G/C-rich sequences, which have been found to be critical regulators of promoter activity [20,21]. The C-rich strand, capable of forming the i-motif (IM) structure, has the following sequence: 5′-TCCTCCTCCTCCTCCTTCTCCTCCTCCTCCGTGACCTCCTCCTCCTCC-3′. Its complementary G-rich strand, capable of forming the G-quadruplex (G4) structure, has the following sequence: 5′-GGAGGAGGAGGAGGTCACGGAGGAGGAGGAGGAGAAGGAGGAGGAGGA-3′ (Table S1). In the present study, circular dichroism (CD) spectroscopy was employed to verify the formation of these secondary structures under our experimental conditions. As shown in Figure S1A, the C-rich sequence exhibited a characteristic positive CD peak at 285–288 nm and a negative peak near 260 nm in the pH range of 5.0–6.25, consistent with i-motif formation [23]. The transitional pH (pH_T_), defined as the pH at which 50% of the C-rich sequences form i-motifs, was determined to be 6.4 through curve fitting (Figure S1B), indicating its relatively high structural stability. Accordingly, a buffer with pH 5.5 was used in our subsequent experiments to ensure complete i-motif formation. In contrast, the CD spectrum of the G-rich sequence exhibited typical features of a hybrid G4 structure (Figure S1C), including positive peaks at 295 nm and 260 nm, as well as a negative peak at 245 nm, indicating that the *c-myb* G4 formed a stable hetero-quadruplex structure at a physiological pH of 7.4 under our experimental conditions [24].

### 2.1. Screening for Binding of Small Molecules to c-myb Promoter Quadruplex Structures

**Figure 1:**
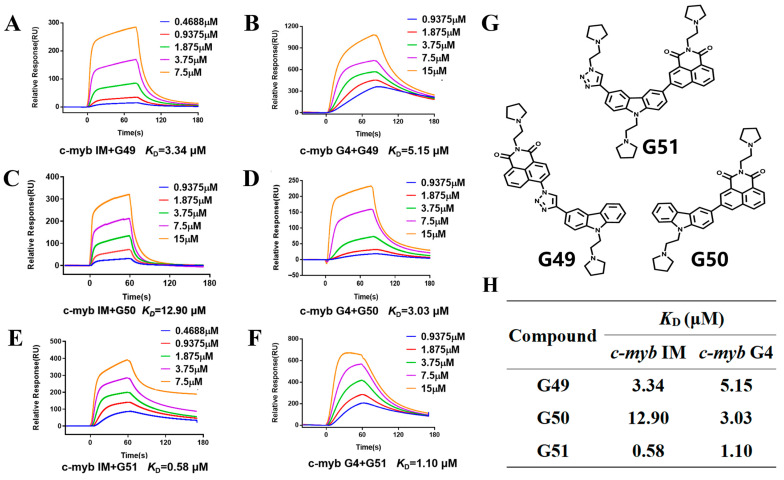
The SPR experiment was performed for the binding of compounds to *c-myb* quadruplex structures. (**A**) The binding of **G49** to *c-myb* i-motif was studied, with the *K_D_* value determined to be 3.34 μM in 1×MES buffer at pH 5.5. (**B**) The binding of **G49** to *c-myb* G4 was studied, with the *K_D_* value determined to be 5.15 μM in Tris-HCl buffer at pH 7.4. (**C**) The binding of **G50** to *c-myb* i-motif was studied, with the *K_D_* value determined to be 12.90 μM in 1×MES buffer at pH 5.5. (**D**) The binding of **G50** to *c-myb* G4 was studied, with the *K_D_* value determined to be 3.03 μM in Tris-HCl buffer at pH 7.4. (**E**) The binding of **G51** to *c-myb* i-motif was studied, with the *K_D_* value determined to be 0.58 μM in 1×MES buffer at pH 5.5. (**F**) The binding of **G51** to *c-myb* G4 was studied, with the *K_D_* value determined to be 1.10 μM in Tris-HCl buffer at pH 7.4. (**G**) Chemical structures of candidate compounds. (**H**) *K_D_* values for binding of candidate compounds to *c-myb* quadruplex structures determined through SPR experiments.

In order to find dual-targeting ligands capable of binding to *c-myb* promoter G-quadruplex and i-motif structures, we screened various compounds, including bisacridine derivatives, acridone derivatives, carbazole derivatives, acridone–naphthalimide hybrid derivatives, and quinoline derivatives, by using surface plasmon resonance (SPR); the data obtained are shown in Table S2. Although most of these compounds had poor binding affinity to *c-myb* i-motif (*K*_D_ > 50 μM), we found that carbazole derivatives **G49** and **G51** showed good binding affinity, with their *K*_D_ values determined to be less than 5 μM. SPR was also performed to assess the binding affinity of these compounds to the *c-myb* G-quadruplex. As shown in Figure 1A,B, **G49** showed moderate binding affinity, with *K*_D_ values determined to be 3.34 μM and 5.15 μM for the i-motif and G4, respectively. In comparison, compound **G50** showed preferential binding to the *c-myb* G-quadruplex only, with the *K*_D_ value determined to be 3.03 μM, without significant interaction with *c-myb* i-motif (Figure 1C,D). Importantly, **G51** exhibited strong binding affinity to both i-motif and G4 structures, with their *K*_D_ values determined to be 0.58 μM and 1.10 μM (Figure 1E,F), respectively. These data suggest that both **G49** and **G51** are potential dual-targeting ligands.

In order to further verify the above data, we studied the binding affinities of **G49**, **G50**, and **G51** to quadruplex structures using microscale thermophoresis (MST). As shown in Figure S2, **G51** exhibited the strongest binding affinity to both i-motif and G-quadruplex structures, with *K*_D_ values determined to be 1.04 ± 0.40 μM for the *c-myb* i-motif and 1.43 ± 0.64 μM for the G4 structure. In comparison, **G49** had a weak binding affinity, with *K*_D_ values determined to be 2.11 ± 0.71 μM for the i-motif and 8.53 ± 3.22 μM for the G4. **G50** exhibited strong binding affinity to the G4 structure, with the *K*_D_ value determined to be 1.11 ± 0.52 μM, without significant interaction with the i-motif (*K*_D_ = 10.06 ± 2.67 μM). Our above data from MST are consistent with our SPR result; therefore, we selected carbazole derivative **G51** (synthetic route as shown in Scheme S1) as the primary compound for further investigation as a dual-targeting ligand for *c-myb* i-motif and G4, with the structurally similar carbazole derivative **G50**, targeting *c-myb* G4 only, as a control for comparison.

### 2.2. Interactions of Selected Carbazole Derivatives with c-myb Promoter Quadruplex Structures

Circular dichroism (CD) spectroscopy, a sensitive technique for monitoring biomolecular conformational changes, was employed to characterize the interactions of our carbazole derivatives with the quadruplex structures [25,26]. As shown in Figure S3, the *c-myb* i-motif alone exhibited characteristic spectral features with a positive peak at 288 nm and a negative peak at 260 nm [23]. Upon the addition of compound **G49** or **G51**, these peaks gradually changed in dose-dependent manners, indicating their good interactions [25,27]. In comparison, compound **G50** had no significant effect on these peaks, indicating their weak interactions. On the other hand, all three compounds affected the positive peak intensity of the *c-myb* G-quadruplex (G4) in dose-dependent manners, indicating their good interactions. These CD experimental data are consistent with our above SPR and MST results.

**Figure 2:**
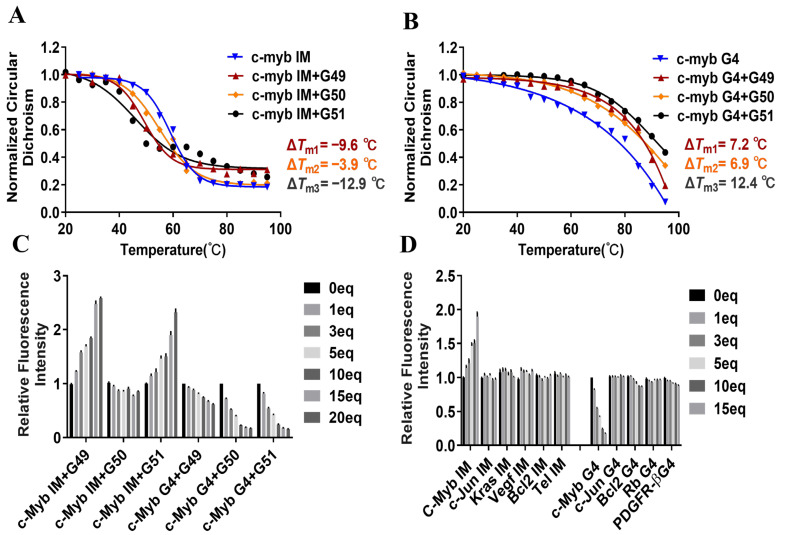
The stabilization effects of carbazole derivatives on *c-myb* promoter i-motif and G-quadruplex structures were studied by using CD melting and FRET experiments. (**A**) The CD melting experiment was performed to analyze CD signal values at 288 nm versus increasing temperatures. *T_m_* values were determined to be 58.9 °C for *c-myb* i-motif alone, 49.3 °C for *c-myb* i-motif with **G49**, 55.0 °C for *c-myb* i-motif with **G50**, and 46.0 °C for *c-myb* i-motif with **G51**. Therefore, *∆T_m_* values were determined to be −9.6 °C for **G49**, −3.9 °C for **G50**, and −12.9 °C for **G51**. (**B**) The CD melting experiment was performed to analyze CD signal values at 265 nm versus increasing temperatures. *T_m_* values were determined to be 80.3 °C for *c-myb* G4 alone, 87.5 °C for *c-myb* G4 with **G49**, 87.2 °C for *c-myb* G4 with **G50**, and 92.7 °C for *c-myb* G4 with **G51**. Therefore, *∆T_m_* values were determined to be 7.2 °C for **G49**, 6.9 °C for **G50**, and 12.4 °C for **G51**. (**C**) The changes in the ratio of fluorescence response values at 518 and 585 nm were determined after the addition of 0, 1, 3, 5, 10, 15, and 20 eq compounds to the *c-myb* i-motif and G4 structure. (**D**) The effects of **G51** on different types of fluorescently labeled DNA secondary structures were studied for the changes in the ratio of fluorescence response values at 518 and 585 nm, with the data normalized and graphed. All experiments were repeated three times in parallel, with data expressed as mean ± SEM.

CD melting experiments were then carried out to evaluate the effects of our carbazole derivatives on the thermal stability of the quadruplex structures [28]. As shown in Figure S4, the intrinsic melting temperatures (T_m_) of our *c-myb* i-motif and G-quadruplex were determined to be 58.9 °C and 80.3 °C, respectively. Upon the addition of **G49**, **G50**, or **G51**, the melting temperature (T_m_) of the i-motif was reduced to 49.3 °C, 55.0 °C, or 46.0 °C, while the melting temperature (T_m_) of the G-quadruplex was increased to 87.5 °C, 87.2 °C, or 92.7 °C, respectively (Figure 2A,B). Therefore, *∆T_m_* values for carbazole derivatives with the i-motif were determined to be −9.6 °C for **G49**, −3.9 °C for **G50**, and −12.9 °C for **G51**, while ∆*T_m_* values for carbazole derivatives with the G-quadruplex were determined to be 7.2 °C for **G49**, 6.9 °C for **G50**, and 12.4 °C for **G51**, respectively. These data showed that **G51** could significantly destabilize the i-motif (Δ*T_m_* = −12.9 °C) while stabilizing G-quadruplex (Δ*T_m_* = +12.4 °C), which was a better dual-targeting ligand than **G49** (Δ*T_m_* = −9.6 °C for i-motif and +7.2 °C for G-quadruplex). In contrast, compound **G50** could stabilize G-quadruplex (Δ*T_m_* = +6.9 °C) without a significant effect on the i-motif (Δ*T_m_* = −3.9 °C). These CD melting experimental data are also consistent with our above binding interaction studies.

In order to further verify the above results, fluorescence resonance energy transfer (FRET) experiments were performed using 5′-FAM and 3′-TAMRA dual-labeled *c-myb* oligomers. The formation of the quadruplex structure can bring the fluorophores into proximity, yielding high FRET efficiency with an emission at 585 nm, while dissociation can reduce this signal [29,30]. As shown in Figure S5C–E, the fluorescence response at 585 nm decreased significantly with increasing concentrations of compounds **G49** and **G51**, indicating that the i-motif structure could be unfolded by these two compounds. In comparison, **G50** showed a very weak unfolding effect on the i-motif. The quantitative analysis (Figure 2C) showed that **G51** could significantly destabilize the i-motif with an increased ratio of fluorescence response values at 518 and 585 nm. On the other hand, as shown in Figure S5F–H, all three compounds, namely, **G49**, **G50**, and **G51**, could stabilize the G-quadruplex with a decreased ratio of fluorescence response values at 518 and 585 nm. Our FRET data are consistent with our above experimental results. All the above results demonstrate that **G51**, as a dual-targeting ligand, could significantly destabilize *c-myb* promoter i-motif while stabilizing *c-myb* promoter G-quadruplex, and in comparison, **G50** could stabilize only *c-myb* promoter G-quadruplex without a significant effect on *c-myb* promoter i-motif.

### 2.3. Carbazole Derivative **G51** Could Specifically Interact with c-myb Promoter Quadruplex Structures Through Destabilization of Its i-Motif and Stabilization of Its G-Quadruplex

Ligand specificity represents a crucial parameter for evaluating small-molecule therapeutics. To assess the binding specificity of compound **G51**, we studied its effect on promoter quadruplex structures from various other oncogenes, including *c-jun*, *HIF-1α*, *Rb*, *Kras*, and *Ret*, by using SPR and MST experiments. As shown in Tables S3 and S4, **G51** exhibited much higher binding affinity to *c-myb* i-motif and G-quadruplex (*K*_D_ = 0.5−1.5 μM) than its binding affinity to other tested secondary structures (*K*_D_ > 7.7 μM), suggesting its excellent specificity. This remarkable specificity was further confirmed by using thiazole orange (TO) displacement assays [31,32], and **G51** showed DC_50_ values of 0.8 μM and 1.0 μM for *c-myb* i-motif and G-quadruplex, respectively (Figure S6). In comparison, **G51** displayed much lower displacement activity on quadruplex structures from other gene promoters, demonstrating its strong binding specificity to *c-myb* promoter quadruplex structures.

CD titration experiments were also performed for the binding of **G51** to some other gene quadruplex structures (Figure S7), which showed that **G51** had no significant effect on these tested quadruplex structures, reinforcing its specific interaction with *c-myb* promoter quadruplex structures. In addition, FRET analysis showed that **G51** had no significant effect on different types of fluorescently labeled DNA secondary structures from promoters of various oncogenes (e.g., *c-Jun*, *Kras*, *VEGF*, and *Bcl-2*), as shown in Figure 2D. We also studied the effect of **G51** on the thermal stability of some other secondary structures from promoters of various oncogenes, including *Hras*, *Bcl-2*, *HIF-1α*, *Ret*, *Rb*, *and c-Jun*, by using a CD melting experiment. As shown in Figure S8, **G51** exhibited no significant impact on these structures, with *ΔT_m_* values ranging from −4.5 to +4.1 °C. These results were further corroborated by using FRET melting experiments, as shown in Figure S9. **G51** had the strongest effect on *c-myb* quadruplex structures, with *ΔT_m_* values determined to be −10.0 °C for the i-motif and +9.3 °C for the G-quadruplex, without a significant impact on other gene quadruplex structures. All the above data suggest that **G51**, as a dual-targeting ligand, had specific binding to *c-myb* promoter quadruplexes, with the destabilization of *c-myb* promoter i-motif and concurrent stabilization of *c-myb* promoter G-quadruplex. This opposing effect could provide a good opportunity for specific gene regulation, with great potential for the development of a precision therapeutic agent.

### 2.4. Further Studies of Interactions Between Carbazole Derivative **G51** and c-myb Promoter Quadruplex Structures

An ultraviolet–visible (UV) experiment was employed to characterize the binding interactions between **G51** and *c-myb* quadruplex structures. Ligand intercalation between DNA base pairs typically manifests through hypochromic effects (reduced absorption intensity) and bathochromic shifts (red shift of absorption maxima), and these phenomena are attributed to π-π stacking interactions [33,34]. As shown in Figure S10, the titration of **G51** with *c-myb* i-motif or G-quadruplex DNA produced hypochromicity, accompanied by red shifts. These spectral perturbations suggest intercalative binding of **G51** to both quadruplex forms, with π-π stacking between the carbazole moiety and nucleobases.

The ESI-MS experiment is a highly sensitive technique capable of detecting non-covalent interactions [35]. As shown in Figure S11, binding of **G51** to *c-myb* i-motif could be detected with the observation of a stable *c-myb* i-motif–**G51** complex (*m*/*z* 13,119.8) in BPES buffer at pH 5.5. In comparison, this complex formation was not observed in BPES buffer at pH 7.0, which indicates that **G51** could bind to the i-motif structure without significant binding to its corresponding C-rich oligomer. Similarly, the binding of **G51** to *c-myb* G4 could also be detected with the observation of a stable *c-myb* G4–**G51** complex (*m*/*z* 14,156.4). These experiments provide direct evidence for the dual binding of **G51** to *c-myb* i-motif and G-quadruplex structures.

We further investigated the interaction of **G51** with *c-myb* promoter quadruplex structures by using an electrophoretic mobility shift (EMSA) experiment [36]. As shown in Figure S12, for the pre-formed i-motif structure, the addition of an increasing amount of **G51** progressively diminished the i-motif band intensity while enhancing the single-strand DNA band, indicating the unfolding of the i-motif structure by **G51**. Conversely, with the unannealed G-rich oligomer, the addition of an increasing amount of **G51** gradually slowed down the migration of the DNA band, indicating G-quadruplex formation induced by **G51**. The above results demonstrate that **G51** could unfold *c-myb* promoter i-motif while inducing G4 formation in concentration-dependent manners. These results provide an in-depth understanding of the opposing effect of **G51** on i-motif and G-quadruplex structures.

### 2.5. **G51** Down-Regulated c-myb Transcription and Translation in HCT116 Cells

**Figure 3:**
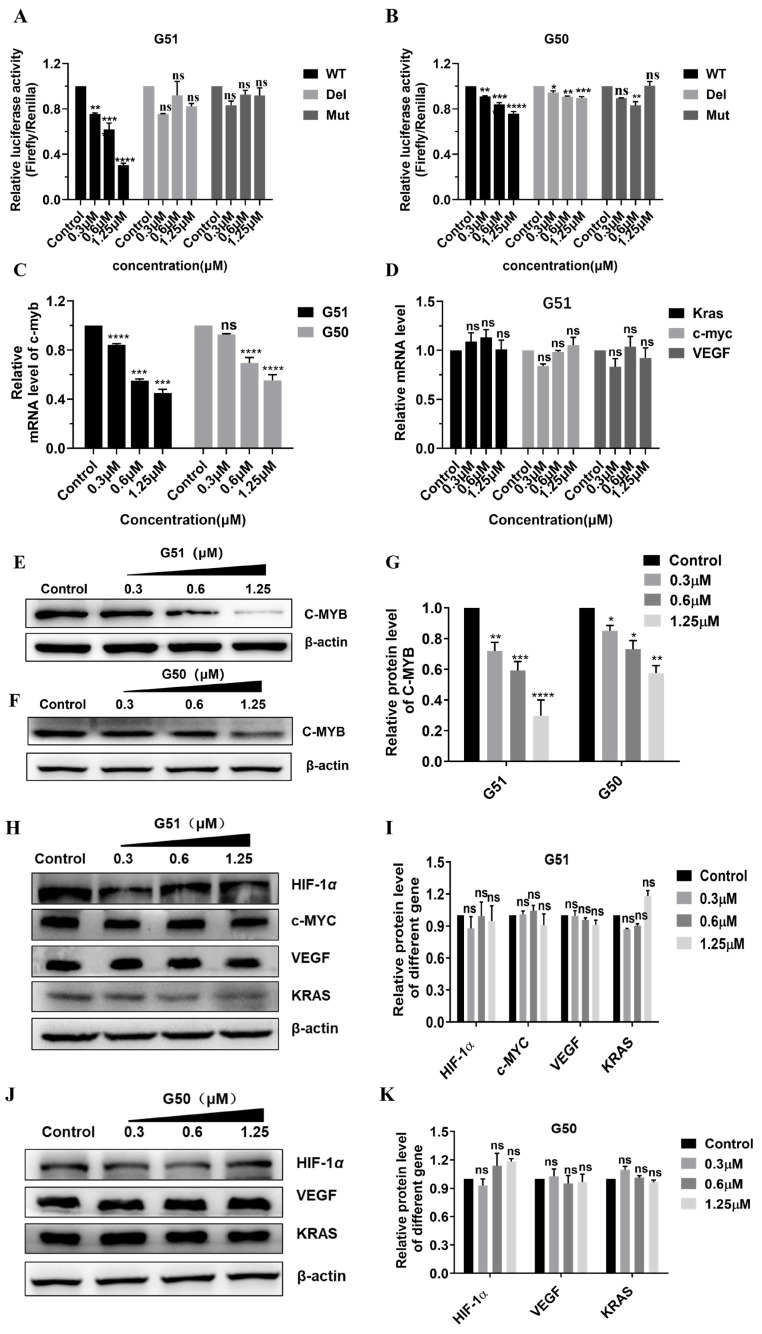
The effects of **G50** and **G51** on *c-myb* transcription and translation were studied by using a dual-luciferase reporter assay, RT-qPCR, and Western blot. (**A**) Effect of **G51** on the expression of luciferase from pGL-WT *c-myb*, pGL-Del *c-myb*, and pGL-Mut *c-myb* plasmid. (**B**) Effect of **G50** on the expression of luciferase from pGL-WT *c-myb*, pGL-Del *c-myb*, and pGL-Mut *c-myb* plasmid. (**C**) The transcription levels of *c-myb* mRNA were affected in HCT116 cells incubated with increasing concentrations of **G50** or **G51** for 48 h. (**D**) The mRNA levels of some other genes were measured for HCT116 cells incubated with **G51** at increasing concentrations for 48 h. (**E**) The effect of **G51** on C-MYB protein expression was analyzed by using a Western blot. (**F**) The effect of **G50** on C-MYB protein expression was analyzed by using a Western blot. (**G**) Histogram of gray value analysis for Western blot bands in Figure (**E**,**F**). (**H**) The effects of **G51** on the expression of some other proteins were analyzed by using a Western blot. (**I**) Histogram of the gray value analysis for Western blot bands in Figure (**H**). (**J**) The effects of **G50** on the expression of some other proteins were analyzed by using a Western blot. (**K**) Histogram of the gray value analysis for Western blot bands in Figure (**J**). The experiments were repeated three times, and the data are shown as mean ± SEM. (ns) not significant, (*) *p* < 0.05, (**) *p* < 0.01, (***) *p* < 0.001, and (****) *p* < 0.0001.

Since **G51** could specifically stabilize *c-myb* promoter G-quadruplex while unfolding its corresponding i-motif structure, we evaluated its regulatory effect on *c-myb* gene transcription and translation. We assessed *c-myb* promoter activity by using a dual-luciferase reporter assay [37] with three engineered recombinant plasmids containing the *c-myb* promoter sequence, including a wild-type *c-myb* plasmid (WT), a deleted *c-myb* plasmid (Del) lacking a quadruplex-forming sequence, and a mutated *c-myb* plasmid (Mut) with a quadruplex-forming sequence mutated to disrupt quadruplex structure formation. These *c-myb* promoter sequences were cloned upstream of firefly luciferase (FLuc) in pGL vectors to construct recombinant plasmids pGL-WT *c-myb*, pGL-Del *c-myb*, and pGL-Mut *c-myb* (Table S6). The plasmid was then co-transfected with Renilla luciferase (RLuc) control plasmid pRL-TK into HCT116 cells. Following 48 h incubation with **G50** or **G51**, dose-dependent inhibition of WT promoter activity was observed, as shown in Figure 3A,B. Notably, **G51** exhibited significantly higher activity than **G50**, indicating that the stabilization of G-quadruplex and destabilization of its corresponding i-motif both contributed positively to the gene regulation. Both compounds had no significant effect on the regulation of the Del or Mut plasmid, indicating their critical interaction with quadruplex structures for regulatory activity.

In order to quantitatively assess the effect of **G51** on *c-myb* transcription, we performed real-time quantitative PCR (qPCR) analysis (primer sequences as shown in Table S7) on HCT116 cells incubated with increasing concentrations of **G50** or **G51** for 48 h. As shown in Figure 3C,D, both compounds exhibited concentration-dependent down-regulation of *c-myb* transcription, and **G51** showed higher regulatory activity than **G50**. Notably, **G51** had no significant effect on the transcription of some other tested genes, including *c-myc*, *VEGF*, and *Kras*, indicating that its regulatory effect on *c-myb* transcription was highly specific. These results also showed that **G51** had higher regulatory activity than **G50**, indicating that the stabilization of G-quadruplex and destabilization of its corresponding i-motif both made positive contributions to the gene regulation.

Next, we further investigated the regulatory effect of **G51** on C-MYB protein translation by using a Western blot (WB) and immunofluorescence (IF) experiments [38]. As shown in Figure 3E–K, both **G50** and **G51** down-regulated C-MYB protein translation in concentration-dependent manners, and **G51** showed much higher regulatory activity than **G50**. In addition, **G51** had no significant effect on the expressions of other proteins, including HIF-1α, C-MYC, VEGF, and KRAS, further confirming its regulatory specificity at the protein translation level. Our immunofluorescence (IF) experiment also showed that the fluorescence intensity of C-MYB protein in HCT116 cells decreased substantially upon incubation with increasing concentrations of **G51** (Figure S13). All these results showed that **G51** could specifically down-regulate *c-myb* expression much more effectively through dual interactions with opposing effects on gene promoter i-motif and G-quadruplex structures.

### 2.6. **G51** Could Induce Cancer Cell Apoptosis with Inhibition of Cell Proliferation and Metastasis

**Figure 4:**
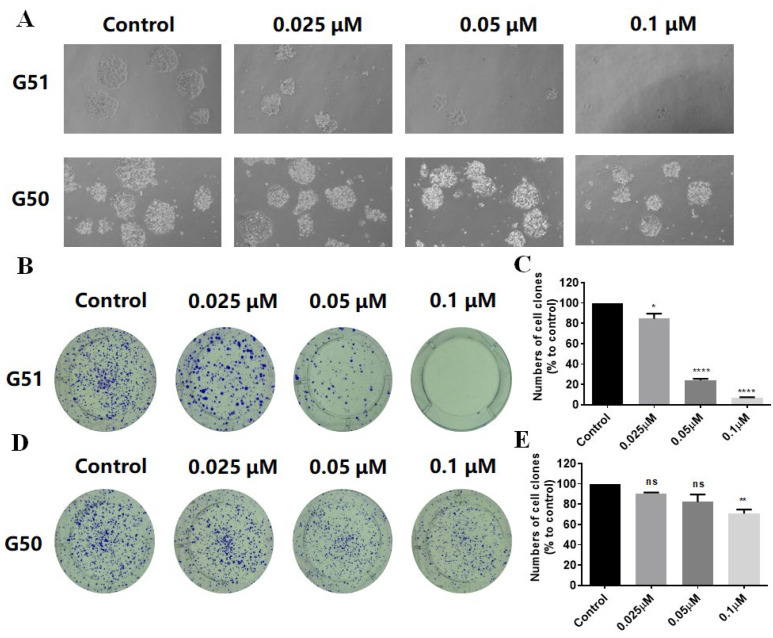
Effects of **G50** and **G51** on colony formation of HCT116 cells. (**A**) Morphological observation of HCT116 cells incubated with compounds for 7 days. (**B**) Representative images of colony formation assay for HCT116 cells incubated with **G51**, with cell numbers quantified as shown in (**C**). (**D**) Representative images of colony formation assay for HCT116 cells incubated with **G50**, with cell numbers quantified as shown in (**E**). Experiments were repeated three times, and data are shown as mean ± SEM, (ns) not significant, (*) *p* < 0.05, (**) *p* < 0.01, and (****) *p* < 0.0001.

The anti-proliferative effects of **G49**, **G50**, and **G51** were evaluated across eight human cancer cell lines (HCT116, SW620, RKO, SW480, DLD1, A549, HGC-27, and HeLa) using an MTT assay, as shown in Table S5. Among these three compounds, **G51** showed the most potent cytotoxicity against almost all tested cancer cell lines, with particular efficacy against HCT116 colorectal cancer cells (IC_50_ = 0.6 ± 0.1 μM). Then, we tested its effect on NCM460 cells (human normal colonic mucosal epithelial cells) for comparison. Our results revealed that **G51** had good selectivity on HCT116 colorectal cancer cells, with only moderate cytotoxicity against NCM460 normal cells (IC_50_ = 12.8 ± 0.5 μM). On the other hand, the effects of **G50** on HCT116 colorectal cancer cells (IC_50_ = 4.7 ± 0.8 μM) and NCM460 normal cells (IC_50_ = 19.8 ± 0.8 μM) were also determined for comparison. These data indicate that **G51** had about five times better selectivity than **G50** on the inhibition of cancer cells versus normal cells, suggesting that the dual-targeting mechanism of **G51** could also decrease off-target side effects. Based on the above data, we selected **G51** as the primary focus for our following in-depth cellular-level studies. To further evaluate the inhibitory effect of **G51** on cell growth, we performed colony formation experiments on HCT116 cells. Our data show that **G51** could reduce the colony number and size, with much higher activity than **G50** (Figure 4). Compound **G51** could significantly reduce the colony number in a dose-dependent manner based on our quantitative analysis, suggesting that **G51** could much more effectively inhibit cell growth, possibly through dual interactions with opposing effects on gene promoter i-motif and G-quadruplex structures.

Then, the effect of **G51** on the apoptosis of HCT116 cells was quantitatively assessed by using the Annexin V-FITC/PI dual-staining method. As shown in Figure S14, dose-dependent increases in apoptosis were observed for HCT116 cells incubated with **G50** or **G51**. Upon treatment with 1.25 μM **G51**, the HCT116 cell apoptosis ratio was increased from 2.65% to 38.32%. In comparison, upon treatment with the same concentration of **G50**, the HCT116 cell apoptosis ratio was increased from 1.81% to 26.61% only. In order to understand the mechanism of HCT116 cell apoptosis induced by **G51**, a Western blot experiment was performed, with the data shown in Figure S15. Our results showed that **G51** also down-regulated BCL-2 expression and up-regulated BAX expression in a dose-dependent manner, which could possibly cause an increase in the permeability of the cell membrane and, consequently, promote the release of cytochrome C from mitochondria. This could trigger the activation of downstream caspase-3 and caspase-9, which could then induce apoptosis. These data are consistent with those for the mitochondrial apoptosis pathway reported previously [39]. Notably, **G51** had a much stronger effect than **G50** on the expression levels of BCL-2 and BAX. Following this experiment, we examined the effect of **G51** on the HCT116 cell cycle by using the propidium bromide (PI) staining method. As shown in Figure S16, upon treatment with **G51**, HCT116 cells in the G_0_/G_1_ phase were significantly increased in a dose-dependent manner, exhibiting a clear G_0_/G_1_ phase block. In comparison, **G50** caused only a slight G_0_/G_1_ phase block. These results demonstrated that **G51**, as a dual-targeting ligand, had potent anti-cancer activity, possibly through the activation of the intrinsic apoptotic pathway and induction of cell cycle arrest at the G_0_/G_1_ phase.

**Figure 5:**
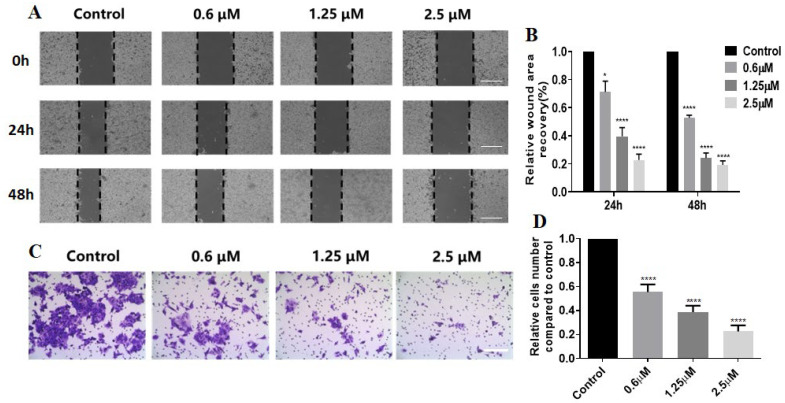
Compound **G51** inhibited the migration of HCT116 cells. (**A**) Cell scratching results for HCT116 cells incubated with increasing concentration of **G51** for 24 h and 48 h (scale bars: 200 μm). (**B**) The wound area recovery data were quantitatively determined and normalized with a control. (**C**) Representative pictures of the transwell assay (scale bar: 200 μm). (**D**) The relative numbers of invaded cells were determined and normalized with a control. The experiments were repeated three times, and the data are shown as mean ± SEM, where * means *p* < 0.05 and **** means *p* < 0.0001.

Next, the effect of **G51** on the migration ability of HCT116 cells was studied through a scratching experiment. As shown in Figure 5A,B, **G51** significantly slowed down the migration of HCT116 cells in a dose-dependent manner upon incubation for 24 h and 48 h, in comparison with the control group. After incubation with **G51** at a 2.5 μM concentration, the scratch area remained almost unchanged, indicating that **G51** could effectively inhibit HCT116 cell migration. We also performed a transwell experiment, as shown in Figure 5C,D. **G51** significantly reduced the number of invaded cells in a dose-dependent manner in comparison with the control group. These experiments show that **G51** could effectively inhibit the migration and invasion of HCT116 cells.

### 2.7. **G51** Inhibited Tumor Growth in a Human Colorectal Cancer Xenograft

**Figure 6:**
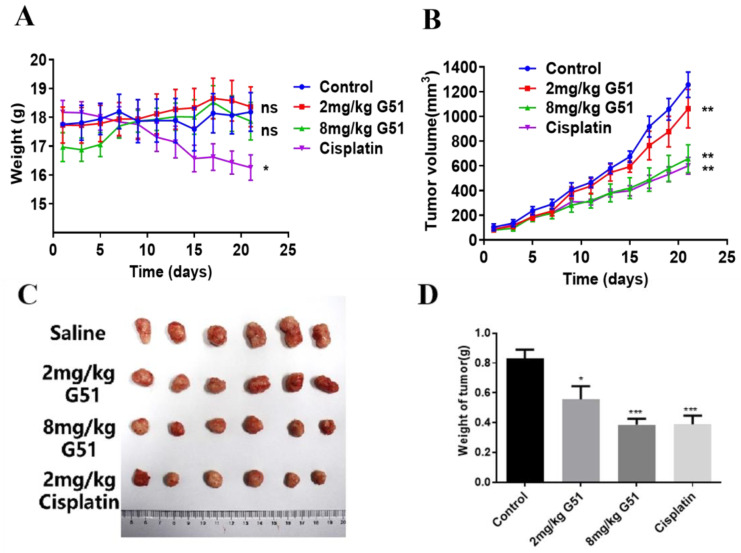
The anti-tumor activity of compound **G51** was analyzed by using an HCT116 xenograft model in nude mice. (**A**) The changes in mouse body weight were measured during the treatment. (**B**) Tumor volumes (mm^3^) of the mice in each group were measured during the treatment. (**C**) Tumor entity images in each group. (**D**) Tumor weights of mice in each group after the treatment. ns means not significant, * means *p* < 0.05, ** means *p* < 0.01, and *** means *p* < 0.001.

Based on the above results, we further evaluated the anti-tumor activity of **G51** in vivo by using an HCT116 xenograft model in nude mice. Tumor establishment was achieved through serial transplantation. The primary tumors grown from 8 × 10^6^ cells were dissected into fragments and re-implanted subcutaneously using a trocar needle. When the tumor volume reached 50–100 mm^3^, the nude mice were randomly divided into four groups (n = 6/group), including a control group (saline), a low-dose group (2 mg/kg **G51**), a high-dose group (8 mg/kg **G51**), and a positive control group (2 mg/kg cisplatin, a platinum-based chemotherapy drug routinely used as a positive control in xenograft models). The drugs were administered intraperitoneally every 48 h for 21 days. Nude mice’s body weight and tumor volume were recorded prior to each administration, as shown in Figure 6A,B. The tumors of the control group continued to grow, while the tumors of the **G51** and cisplatin groups grew at a significantly slower rate. As shown in Figure 6C,D, the tumor growth inhibition ratio (TGI) was calculated using the equation (1—tumor weight in drug-treated group/tumor weight in saline group) to evaluate the inhibitory effect of the drug. The TGI values were determined to be 31% for the low-dose **G51** group, 54% for the high-dose **G51** group, and 56% for the cisplatin group, which indicates that **G51** could inhibit tumor growth in a dose-dependent manner. TGI analysis revealed comparable efficacy between high-dose **G51** (54%) and cisplatin (56%), demonstrating the potent in vivo anti-tumor activity of **G51**. Importantly, no significant body weight changes were observed in **G51** treatment groups in comparison to the cisplatin group, which had obvious body weight loss (Figure 6A), suggesting no significant side effect for **G51** treatment at therapeutic doses.

**Figure 7:**
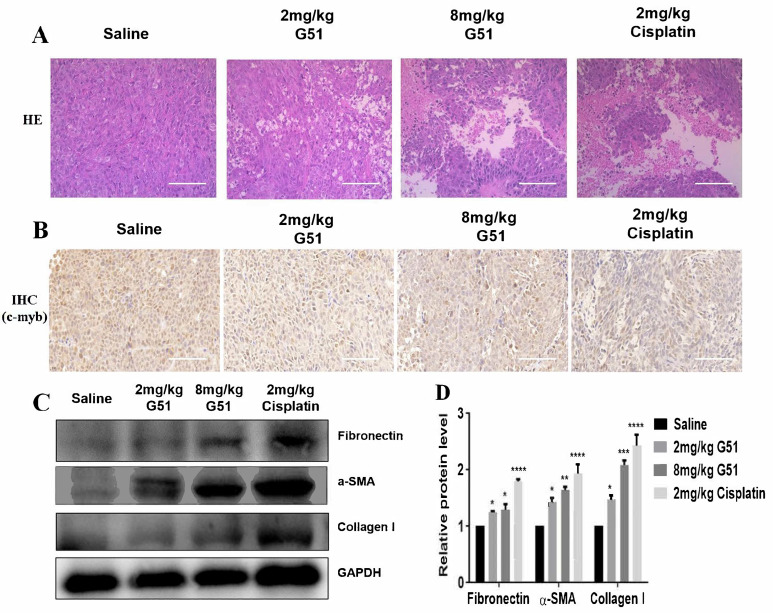
Effect of **G51** on liver and tumor tissues. (**A**) HE-stained images of tumor tissues in different treatment groups (scale bars: 200 μm). (**B**) Images of tumor immunohistochemistry (scale bars: 200 μm). (**C**) Expressions of inflammatory factors in the livers of mice. (**D**) The experiments were repeated three times, and the data are shown as mean ± SEM. (*) *p* < 0.05, (**) *p* < 0.01, (***) *p* < 0.001, and (****) *p* < 0.0001.

To further understand the anti-tumor mechanism of compound **G51**, we performed hematoxylin–eosin (HE) staining and immunohistochemical (IHC) analysis of tumor tissues. As shown in Figure 7A, HE staining revealed distinct morphological differences between the saline control group and treatment groups. The cells in the saline group exhibited tightly packed and uniform cells with intact morphology, while the **G51** treatment groups showed a dose-dependent increase in necrotic areas, characterized by cellular shrinkage, nuclear fragmentation, and loss of membrane integrity. These pathological changes were comparable to those observed in the cisplatin group, which indicates that **G51** could induce necrosis of tumor tissues with a similar effect to that of cisplatin. Meanwhile, IHC analysis, as shown in Figure 7B, demonstrated that the expressions of C-MYB protein in the tumor cells, as indicated with brown dots for both **G51** treatment groups and the cisplatin group, were significantly decreased in comparison with the saline control group, confirming that **G51** was also able to down-regulate *c-myb* in in vivo models, consistent with our in vitro Western blot experimental data.

In addition to its anti-tumor activity, we evaluated the toxicity of **G51** for safety reasons. As shown in Figure 6A, the body weights of mice in the **G51** treatment groups remained stable throughout the experiment, while mice in the cisplatin group exhibited apparent weight loss, indicating that long-term cisplatin administration induced systemic side effects. To assess drug safety, we also performed HE staining to examine cellular morphology in major organs (heart, liver, spleen, lung, and kidney) of nude mice. As shown in Figure S17B, no significant pathological lesions were observed in the **G51** treatment groups and the cisplatin group in comparison to the saline control group. Since hepatic fibrosis is a critical marker of liver injury and its irreversibility can lead to cirrhosis, we further evaluated the hepatic toxicity of **G51** by analyzing the expression of fibrosis markers, including Fibronectin, α-SMA, and Collagen I [40]. As shown in Figure 7C,D, both **G51** treatment groups and the cisplatin group exhibited elevated expressions of Fibronectin, α-SMA, and Collagen I in comparison to the saline control group. However, their increases in the **G51** treatment groups were much lower than those in the cisplatin group, indicating that **G51** did not induce significant liver toxicity. In addition, as shown in Figure S17A, organ weight analysis showed reduced organ weights in the cisplatin group, while organ weights in the **G51** treatment groups had no significant changes, further supporting its favorable safety profile. All the above results demonstrate that **G51** exhibits lower toxicity than cisplatin, highlighting its potential advantage for further development for clinical application.

In summary, these results demonstrate that **G51** exhibits potent anti-tumor activity in human colorectal cancer xenograft models at experimental doses, without apparent toxicity to major organs, highlighting its potential as a lead compound for further development of anti-cancer agents. Our findings reveal that compound **G51**, as a dual-targeting ligand, derives its anti-tumor effects through specific i-motif destabilization coupled with G-quadruplex stabilization. This discovery sets up a novel foundation for developing anti-cancer drugs through coordinated regulation of gene quadruplex structures.

## 3. Discussion

In this study, we identified and validated carbazole derivative **G51** as a dual-targeting ligand simultaneously binding to both i-motif and G-quadruplex on the *c-myb* promoter region. **G51** could significantly down-regulate *c-myb* transcription and translation through opposing effects on different quadruplex structures, with potent anti-tumor activity. In comparison, carbazole derivative **G50**, which only targets the G-quadruplex, showed very weak anti-tumor activity, highlighting the therapeutic advantage of dual-targeting ligands. Our comprehensive in vitro and in vivo experiments demonstrated that **G51** could effectively inhibit tumor cell proliferation, migration, and invasion. **G51** could also induce cancer cell apoptosis, according to our cell cycle analysis. Importantly, **G51** exhibited potent anti-tumor activity in our HCT116 xenograft model, without significant side effects in comparison to the conventional chemotherapeutic agent cisplatin. This study not only expands the therapeutic potential of carbazole derivatives but also provides a novel example for developing anti-cancer drugs through coordinated opposing effects on different quadruplex structures for the specific regulation of gene expression.

Our present study demonstrated that **G51** could become a promising lead compound for further development for colorectal cancer treatment. While this study provides promising results, some aspects could be further investigated in the future. The precise binding modes or interactions between **G51** and quadruplex structures could be illustrated through structural studies of the binding complex using X-ray crystallography or cryo-electron microscopy for guiding rational ligand optimization. Due to the weak fluorescence activity of **G51**, direct visualization of its intracellular target engagement remains challenging. Its structural modifications through fluorophore conjugation or molecular optimization could enable real-time visualization of intracellular interactions to validate target specificity and mechanisms. The potential crosstalk between i-motif destabilization and G-quadruplex stabilization could also be studied for clarification. Systematic mutagenesis, combined with single-molecule imaging approaches, could determine whether these effects operate independently or synergistically. The comprehensive evaluation of the pharmacokinetic properties of **G51**, including its absorption, distribution, metabolism, and excretion (ADME), along with a detailed toxicological assessment in multiple animal models, could also be performed for future drug development.

In the present study, our primary focus was to investigate the effect of **G51** on regulating *c-myb* transcription and translation through opposing effects on different quadruplex structures on the gene promoter for cancer treatment. The study of the effect of **G51** could be further expanded to some other potential targets. Since **G51** could stabilize *c-myb* promoter G-quadruplex, it would be interesting to further study its effect on telomeric G-quadruplex, which could possibly induce DNA damage, especially for telomeres. This aspect could be investigated by performing immunofluorescence combined with fluorescence in situ hybridization (IF/IF-FISH) analysis to assess the induction of DNA damage using gH2AX as a DNA damage marker for foci formation and its localization at telomeres. **G51** could be compared to **G50** for their DNA damage effects to investigate if the specificity of **G51** for *c-myb* alters the capability of the compound to induce telomeric DNA damage.

In addition, direct evidence confirming i-motif interaction within a cellular context could be studied in the future. For instance, chromatin immunoprecipitation with quadruplex-specific antibodies or structure-specific chemical foot-printing in cells could further substantiate the proposed mechanism. However, the lack of reliable sources for i-motif-specific antibodies and the technical challenges of probing transient non-canonical structures in intact cells currently prevent these experiments from being carried out by our group. In addition, the selectivity of **G51** for the *c-myb* promoter over other genomic quadruplex-forming sequences could also be investigated in the future. Although quadruplex structures are prevalent in multiple oncogene promoters, the observed low in vivo toxicity suggests high specificity with tolerable off-target effects.

In summary, this study demonstrates that the carbazole derivative **G51** significantly inhibits *c-myb* expression and exerts potent anti-tumor activity by simultaneously dismantling the i-motif and stabilizing the G-quadruplex in the *c-myb* promoter region. **G51** represents a promising anti-tumor lead compound capable of bidirectionally modulating the stability of i-motif and G-quadruplex structures in the *c-myb* promoter. This bidirectional modulation capability is potentially significant because it could overcome the compensatory effects usually associated with single-target approaches, where the inhibition of one target structure could lead to up-regulation or compensation through the other. This study also provides a novel example for coordinated gene regulation through multiple non-canonical DNA structures, which reveals that the *c-myb* promoter i-motif and G-quadruplex can be co-targeted for synergistic effects. This discovery not only provides novel insights for developing anti-tumor drugs targeting nucleic acid secondary structures but also offers promising new strategies for cancer therapy through coordinated gene promoter quadruplex modulation. While further optimization and investigation are required, this work establishes an important theoretical and experimental foundation for developing dual-targeting ligands as potent anti-tumor lead compounds with low toxicity and few side effects.

## 4. Materials and Methods

### 4.1. Oligonucleotides and Compounds

The nucleotide oligomers used in this study were purchased from Sangong (Shanghai, China). Prior to use, the oligomers were centrifuged at 6000× *g* rpm for 2 min, dissolved in ultra-pure water to a concentration of 100 μM with a vortex, and then stored at −20 °C. For the experiments, the oligomers were diluted with a buffer to the required concentrations. All oligomer sequences are listed as shown in Table S1. The DNA oligonucleotides were subjected to thermal annealing (95 °C, 10 min) followed by a controlled cooling ramp (1 °C/min) to facilitate the formation of secondary structures, which were then stored at 4 °C for later usage. Their relevant working solutions were prepared through dilution with appropriate buffers, which are specified in Table 1.

The compounds used for screening have been previously synthesized in our laboratory, including bisacridine derivatives, acridone derivatives, acridone–naphthimide derivatives, carbazole derivatives, and quinoline derivatives, with their structures shown in Table S2. The compounds were dissolved in DMSO as a 10 mM reservoir solution and stored at −20 °C in a freezer.

### 4.2. Cell Culture

All types of cells were originally purchased from Procell Life Science & Technology Co., Ltd. (Wuhan, China) and were cultured in our laboratory using an ESCO constant-temperature incubator at 37 °C with 5% CO_2_. These included cervical adenocarcinoma cells (HeLa), non-small cell lung cancer cells (A549), gastric cancer cells (HGC-27), and human colon cancer cells (HCT-116, SW620, SW480, RKO, and DLD-1). The cell culture consisted of an RPMI-1640 (or DMEM or MEM) basic medium, supplemented with 10% fetal bovine serum (FBS) and 1% penicillin/streptomycin. Specifically, HCT-116 and HGC-27 cells were cultured in an RPMI-1640 medium with 10% FBS, while Siha, A549, SW480, HepG2, and U2OS cells were cultured in a DMEM medium containing 10% FBS. HeLa cells were cultured in an MEM basic medium supplemented with 10% FBS.

### 4.3. Circular Dichroism (CD) Spectroscopy and CD Melting Experiment

The oligomers for i-motif and G-quadruplex (G4) were diluted to 2 μM with a BPES buffer (for i-motif) and Tris-HCl buffer at pH 7.4 (for G4), respectively, and stored at 4 °C after annealing. CD spectra from 230 to 350 nm were recorded on a Chirascan^®^ circular dichroism spectrophotometer (Applied Photophysics, Leatherhead, UK) with a 10 mm path length quartz cuvette. The buffer was scanned at first, as a blank, followed by the scanning of the i-motif or G4-containing solution. After the addition of the compounds, the mixture was incubated with a vortex for 2–3 min, followed by scanning under the same conditions. For CD melting experiments, scanning was performed at a temperature range of 25–95 °C, with an increasing rate of 1 °C/min, and recorded at 5 °C intervals. The CD signals at 288 nm for i-motif and 265 nm for G4 were plotted against temperature using GraphPad Prism 9.0, and T_m_ values were determined. All spectra were blank-subtracted using corresponding buffer controls.

### 4.4. Surface Plasmon Resonance (SPR) Experiment

All buffers and regeneration reagents were filtered through a 0.22 μm membrane. Molecular interactions were characterized using a Biacore X100 system (Cytiva, Waltham, MA, USA) controlled by Biacore-8K Software v5.0.18. Streptavidin (1 mg/mL in sodium acetate, pH 5.0) was covalently immobilized on CM5 sensor chips. A biotin-labeled i-motif or G4 oligomer, with a sequence as shown in Table S1, was diluted to 1 nM with MES buffer at pH 5.5 or Tris-HCl buffer at pH 7.4, annealed, and stored at 4 °C overnight. The annealed oligomer with a certain secondary structure was then immobilized on the CM5 chip. The compound was serially diluted with the corresponding buffer to generate a concentration gradient, with the DMSO content fixed at 5%. The following instrument parameters were used, including a flow rate of 30 μL/min, binding time of 60 s, and dissociation time of 100 s. Regeneration was performed using 1.5 M glycine and 1 M KCl. The binding constant (K_D_) was determined by fitting the data with Biacore Insight Evaluation 5.0.18 software.

### 4.5. Microscale Thermophoresis (MST) Experiment

A 5′-FAM-labeled DNA oligomer was diluted to 2 μM with MES buffer at pH 5.5 or Tris-HCl buffer at pH 7.4, annealed to form a stable i-motif or G-quadruplex structure, and serially diluted (0.25–2 μM) using a two-fold dilution method. The following instrument settings were used: 40% LED blue intensity and 40% MST power. The compound was two-fold diluted to 16 concentrations starting from 2000 μM, mixed at a 1:1 volume ratio with DNA (5 μL each), and incubated for 10 min before MST analysis. The binding constant (K_D_) was derived using NTAnalysis 1.5.41 software.

### 4.6. TO Displacement Experiment

The i-motif/G4-forming oligonucleotides in 2 μM BPES at pH 5.5 and Tris-HCl at pH 7.4, respectively, were annealed and stored at 4 °C. Samples were mixed and stained with thiazole orange (TO) at a final concentration of 2 μM for 30 min without light. Fluorescence was measured (λ_ex_ = 480 nm, λ_em_ = 500–700 nm) after the incremental addition of 1 equivalent compound per step until signal plateau. The displacement ratio was calculated based on the response at 530 nm using Origin and GraphPad 9.0.

### 4.7. Fluorescence Resonance Energy Transfer (FRET) Experiment

A 5′-FAM/3′-TAMRA-labeled oligomer for i-motif or G4 (0.1 μM in BPES buffer at pH 5.5 or Tris-HCl buffer at pH 7.4) was annealed. Fluorescence profiles with excitation at 480 nm and an emission scan in the range of 500–700 nm were measured after the addition of the compound at 1–15 eq. FRET efficiency was determined with the ratio of 518 nm (FAM) to 585 nm (TAMRA) fluorescence data. For the FRET melting experiment, the temperature was ramped up from 25 to 95 °C at a rate of 1 °C/min, while fluorescence was measured at 1 °C intervals, with T_M_ and ΔT_M_ derived using GraphPad.

### 4.8. Electrophoretic Mobility Shift Assay (EMSA) Experiment

A *c-myb* promoter oligomer for i-motif or G4 was prepared at 5 μM with BPES buffer at pH 5.5 or Tris-HCl buffer at pH 7.4, respectively, and then annealed. The oligomer was incubated with different equivalents of the compound for 24 h. Electrophoresis was carried out for 3–4 h on ice with a 10% non-denaturing gel at a voltage of 80 V and at pH 5.5 or 7.4. The gel was silver-stained and photographed after electrophoresis.

### 4.9. Ultraviolet–Visible (UV) Spectroscopy

A *c-myb* promoter oligomer for i-motif or G4 was prepared at 1000 μM with BPES buffer at pH 5.5 or Tris-HCl buffer at pH 7.4, respectively, and then annealed. The **G51** solution was prepared at a 1 μM concentration for subsequent UV scanning in the range of 350–550 nm. The *c-myb* oligomer for i-motif or G4 was added dropwise so that its final concentration was increased in increments of 5 μM. The solution was incubated for 2 min after each addition and then analyzed with UV spectroscopy.

### 4.10. ESI-MS

A *c-myb* promoter oligomer for i-motif or G4 was prepared at 5 μM with BPES buffer at pH 5.5 or Tris-HCl buffer at pH 7.4, respectively, and then annealed. The oligomer was mixed with 10 eq **G51**, and the mixture was incubated for 24 h, which was then analyzed using an LCQ DECA PLUS XP mass spectrometer (Thermo Fisher Scientific, Waltham, MA, USA).

### 4.11. Dual-Luciferase Reporter Assay

For the dual-luciferase reporter assay, HCT116 cells were seeded in 96-well plates at a density of 5 × 10^3^ cells per well (triplicate wells) and allowed to adhere overnight. Cells were transfected for 4–5 h with 100 ng each of pGL-WT *c-myb* (or pGL-Del *c-myb* or pGL-Mut *c-myb*) plasmid, along with the pRL-TK control plasmid, using the Lipo8000™ (Beyotime, Shanghai, China) transfection reagent. Following transfection, the cells were treated with the serially diluted compound **G51** or **G50** in an RPMI-1640 medium for 48 h. The cells were lysed, and R-Luc and F-Luc reaction solutions were added. Luciferase activity was detected, and the data were analyzed using Graphpad 9.0.

### 4.12. Reverse Transcription–Quantitative Polymerase Chain Reaction (RT-qPCR)

HCT116 cells were seeded in 6-well plates at a density of 1.6 × 10^5^ cells per well and allowed to adhere overnight. The cells were treated with a serially diluted compound in an RPMI-1640 medium for 48 h. Total RNA was extracted from each well using 1 mL AG RNAex Pro Reagent (Acres Biological, Changsha, China, #AG21101). Reverse transcription was performed using Evo M-MLV (Acres Bio, Changsha, China, #AG11706), followed by qPCR using the SYBR Green Pro Taq HS Premix (GenStar, Guangzhou, China). Each experiment was performed in triplicate. Data analysis and visualization were conducted using GraphPad Prism 9.0.

### 4.13. Western Blot

HCT116 cells were seeded in 6-well plates at a density of 1.6 × 10^5^ cells per well and allowed to adhere overnight. The cells were treated with a serially diluted compound in an RPMI-1640 medium for 48 h. Following treatment, the cells were lysed with RIPA (Bioteke, Wuxi, China) buffer on ice for 30 min. Protein quantification was performed using a BCA assay (Thermo Fisher, Waltham, MA, USA). Aliquots (30 μg/lane) were resolved by using SDS-PAGE and transferred to PVDF membranes (0.22 μm). After blocking, membranes were probed with primary antibody (1:1000, 4 °C, overnight) and HRP-conjugated secondary antibody (1:3000, RT, 50 min). Chemiluminescent signals were captured using a Tanon-4200SF imaging system (Tanon Science & Technology Co., Ltd., Shanghai, China).

### 4.14. Immunofluorescence

HCT116 cells (5 × 10^3^/well) were cultured overnight in 96-well plates before treatment with serially diluted **G51** in RPMI-1640 for 48 h. Fixed cells (4% PFA) were permeabilized (0.5% Triton X-100), blocked with 5% BSA, and immunostained with anti-c-myb (4 °C, overnight), followed by Alexa Fluor 488-conjugated secondary antibody (#A21206, Life Technologies, Carlsbad, CA, USA) at 37 °C for 30 min. Nuclei were counter-stained with DAPI (Invitrogen, Carlsbad, CA, USA) prior to imaging (Olympus FV3000 confocal microscope, Tokyo, Japan).

### 4.15. Cytotoxic Assay

Cell suspensions (HCT116 and other types of cells) were plated in 96-well microplates (5 × 10^3^ cells/well) and cultured for 24 h to ensure attachment. Test compounds were prepared through logarithmic dilutions (20−0.3125 μM final concentration) in a medium and applied to cells for 48 h exposure. The activity was determined by using an MTT assay through 4 h incubation with 0.5 mg/mL methylthiazolyldiphenyl-tetrazolium bromide at 37 °C. Following the solubilization of formazan products in DMSO, optical density measurements were recorded at 570 nm using a Bio-Tek microplate reader (Winooski, VT, USA). Triplicate determinations were performed for each condition, with dose–response curves generated using nonlinear regression in GraphPad Prism (v9.0).

### 4.16. Colony Formation Assay

For the long-term proliferation assessment, cells were seeded at a low density (1 × 10^3^ cells/well) in 6-well plates and exposed to **G51**/**G50** (0.1−0.025 μM) for 7 days with medium replacement every 48 h. After methanol fixation and 0.1% crystal violet staining, colonies (>50 cells) were enumerated using automated image analysis.

### 4.17. FITC Annexin V/PI Cell Apoptosis Analysis

Using 6-well plates (1 × 10^5^ cells/well), HCT116 cultures were treated with **G51**/**G50** (1.25−0.3 μM) for 24 h. Dual staining with Annexin V-FITC and propidium iodide using an Apoptosis Kit (#AP101-100-kit, Multi Sciences, Hangzhou, China) was performed according to the manufacturer’s specifications. Quantitative analysis of apoptotic populations was conducted on a CytoFLEX S flow cytometer (Beckman Coulter, Brea, CA, USA), with compensation controls using single-stained samples.

### 4.18. Cell Cycle Experiments

Following 24 h exposure to test compounds (0.6−0.15 μM), ethanol-fixed HCT116 cells were treated with RNase A (100 μg/mL) and propidium iodide (50 μg/mL) for 30 min at 37 °C. The CytoFLEX S flow cytometer (Beckman Coulter, Brea, CA, USA) was used to analyze cell cycle distribution.

### 4.19. Cell Scrape Assay

HCT116 cells were seeded in 6-well plates at a density of 1 × 10^6^ cells per well and allowed to adhere overnight. A uniform scratch wound was created using a 10 μL pipette tip, followed by replacement with a serum-free RPMI-1640 medium. The cells were treated with the serially diluted compound **G51** at a concentration range of 2.5–0.6 μM in a serum-free medium for 48 h. Wound closure was monitored and photographed after 0, 24, and 48 h using phase-contrast microscopy.

### 4.20. Transwell Assay

Matrigel matrix was diluted 1:8 with a serum-free RPMI-1640 medium and coated onto the upper surface of transwell chambers. HCT116 cells (8 × 10^4^ cells per chamber) were seeded in the coated chambers. The cells were treated with the serially diluted compound **G51** at a concentration range of 2.5–0.6 μM in a serum-free medium for 24 h. Following incubation, the cells were fixed with 4% paraformaldehyde and stained with 0.1% crystal violet. Invaded cells were photographed using an EVOS FL Auto imaging system (Thermo Fisher Scientific, Waltham, MA, USA).

### 4.21. Evaluation of In Vivo Anti-Tumor Activity

SPF-grade male BALB/c-nu/nu nude mice (3–4 weeks old) were obtained from the Laboratory Animal Center of Sun Yat-Sen University. The mice were housed under controlled conditions (22 ± 1 °C, 60–70% humidity, and 12 h light/dark cycle). All experimental procedures were approved by the Institutional Animal Care and Use Committee of Sun Yat-Sen University (Approval No. SYSU-IACUC-2023-001980; Approval date: 19 September 2023) and complied with national guidelines for laboratory animal welfare.

For tumor xenografts, 200 μL of an HCT116 cell–Matrigel mixture (8 × 10^6^ cells/mouse) was subcutaneously inoculated into the right shoulder using a sterile 1 mL syringe. Tumor volume was monitored, with data recorded every other day. When tumors reached 50–100 mm^3^ (approximately 8 days post-implantation), the mice were randomly allocated into four groups, including a control group (saline), a low-dose group (2 mg/kg **G51**), a high-dose group (8 mg/kg **G51**), and a positive control group (2 mg/kg cisplatin). Treatments were administered via intraperitoneal injection every other day, with body weights and tumor measurements recorded prior to each administration.

At the experimental endpoint (control tumor volume = 1400 mm^3^), the mice were euthanized, and their organs (including hearts, livers, spleens, lungs, and kidneys) and tumors were collected. Tissues were either bisected, followed by storage at −80 °C, or fixed in 4% paraformaldehyde. Tumor inhibition ratios were calculated as follows: IR = [1 − (mean experimental tumor weight/mean control tumor weight)] × 100%. Fixed tissues were processed for IHC and H&E staining by Servicebio company (Wuhan, China), with imaging obtained using a digital pathology system. Western blotting was performed to analyze Fibronectin, α-SMA, and Collagen I expressions in liver tissues.

### 4.22. Statistical Analysis

Data are presented as mean ± SEM. Statistical comparisons between groups were performed using either one-way ANOVA or unpaired Student’s *t*-tests in GraphPad Prism (GraphPad Software Inc., La Jolla, CA, USA). A *p*-value of ≤0.05 (*) was considered statistically significant.

## Figures and Tables

**Table 1:** Composition of experimental buffers.

Buffer	Ingredients
MES buffer	20 mM MES and 100 mM KCl at pH 5.5
BPES buffer	30 mM (KH_2_PO_4_, K_2_HPO_4_), 100 mM KCl, and 1 mM EDTA
Tris-HCl buffer	50 mM Tris-HCl and 100 mM KCl at pH 7.4

## Data Availability

The data presented in this study are available in both the article and the Supplementary Materials.

## Supplementary Materials

The following supporting information can be downloaded at: https://www.mdpi.com/article/10.3390/ijms26178299/s1.
