# Supplementary material for: Down-Regulation of Oncogene c-myb Specifically by Carbazole Derivative Through Opposing Effects on Different Quadruplex Structures of Gene Promoter for Cancer Treatment
Source: Int J Mol Sci. 2025 Aug 27;26(17):8299. doi: 10.3390/ijms26178299 (PMC12427763; doi:10.3390/ijms26178299)
Supplement: Supplementary file 1 [file ijms-26-08299-s001.zip › ijms-3807773-supplementary.pdf]

Supplementary Data for

Down-Regulation of Oncogene *c-myb* Specifically by Carbazole Derivative Through Opposing Effects on Different Quadruplex Structures of Gene Promoter for Cancer Treatment

Siyi Wang†, Jihai Liang†, Jiahui Zhang, Dongsheng Ji, Zhi-Shu Huang, and Ding Li*

School of Pharmaceutical Sciences, Sun Yat-sen University, Guangzhou University City, Guangzhou 510006, P. R. China

† These authors contributed equally to this work

* Corresponding author: Ding Li, Tel: 8620 3994 3058; E-mail: liding@mail.sysu.edu.cn

Table of Contents

**Figure S1.** CD experiment showed that *c-myb* gene promoter could form i-motif and G-quadruplex structures	**S4**
**Figure S2.** MST experiments were performed for binding of compounds to *c-myb* quadruplex structures	**S4**
**Figure S3.** Effects of **G49, G50, G51** on *c-myb* promoter quadruplex structures were studied by using CD	**S5**
**Figure S4.** The thermodynamic stabilities of *c-myb* i-motif and G4 structures were measured by using CD melting spectra	**S6**
**Figure S5.** Fluorescence response experiments were carried out to study the effects of compounds on the formation of *c-myb* promoter quadruplex structures	**S7**
**Figure S6.** TO displacement experiment for quadruplexes	**S8**
**Figure S7.** CD experiment was performed for binding of **G51** to other gene quadruplex structures	**S9**
**Figure S8.** CD melting experiments were carried out to study the effect of **G51** on stabilizing various DNA secondary structures	**S10**
**Figure S9.** ΔTm values for various DNA secondary structures interacting with compound **G51** were determined by using FRET melting	**S11**
**Figure S10.** UV titration experiment was performed to study the interaction of **G51** with *c-myb* promoter quadruplex structures	**S11**
**Figure S11.** ESI-MS spectra of *c-myb* promoter C-rich (Py42) and G-rich (Pu42) oligomer with or without addition of **G51**	**S12**
**Figure S12.** EMSA experiment was performed with silver staining for the effect of **G51** on *c-myb* promoter quadruplex structures	**S13**
**Figure S13.** The expression of C-MYB protein in HCT116 cells incubated with **G51** for 48h was analyzed by using immunofluorescence	**S13**
**Figure S14.** The effects of **G51** and **G50** on apoptosis of HCT116 cells	**S14**
**Figure S15.** Effects of compounds **G51** and **G50** on the expressions of apoptosis-related proteins in HCT116 cells	**S15**
**Figure S16.** Cell cycle analysis of HCT116 cells upon treatment with compounds **G51** and **G50** for 24h	**S15**
**Figure S17.** Comparison of vital organs for mice in different treatment groups	**S15**
**Scheme S1.** Synthetic pathway for carbazole derivative **G51**	**S16**
**Table S1.** Oligonucleotides used in this study	**S16**
**Table S2.** Equilibrium binding constants (K_D) determined by using SPR	**S18**
**Table S3.** Binding affinity of **G51** to different i-motifs and G4s determined by using	**S22**

SPR	
**Table S4.** Binding affinity of **G51** to different i-motifs and G4s determined by using MST	**S23**
**Table S5.** IC_{50} (μM) values were determined for effects of **G49**, **G50** and **G51** on various types of cells by using MTT assay	**S23**
**Table S6.** The oligonucleotides for wild type or deleted *c-myb* promoter used for pGL-3 Basic plasmids, with underlined text indicating quadruplex forming sequence or mutant sequence	**S24**
**Table S7.** Primers used for qRT-PCR	**S24**
^{1}H NMR and ^{13}C NMR spectra of carbazole derivatives	**S25**

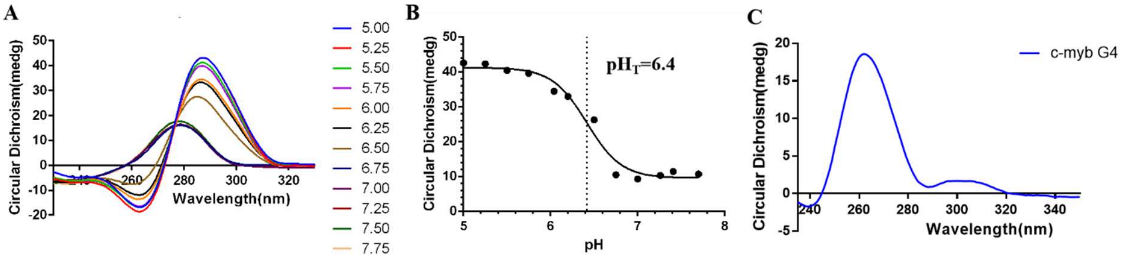

Figure S1. CD experiment showed that *c-myb* gene promoter could form i-motif and G-quadruplex structures. (A) CD spectrum of *c-myb* promoter i-motif in BPES buffer at different pH. (B) CD spectrum value at 288 nm for *c-myb* promoter i-motif against pH was used to determine pH_T through curve fitting. (C) CD spectrum of *c-myb* promoter G-quadruplex in Tris-HCl buffer at pH 7.4.

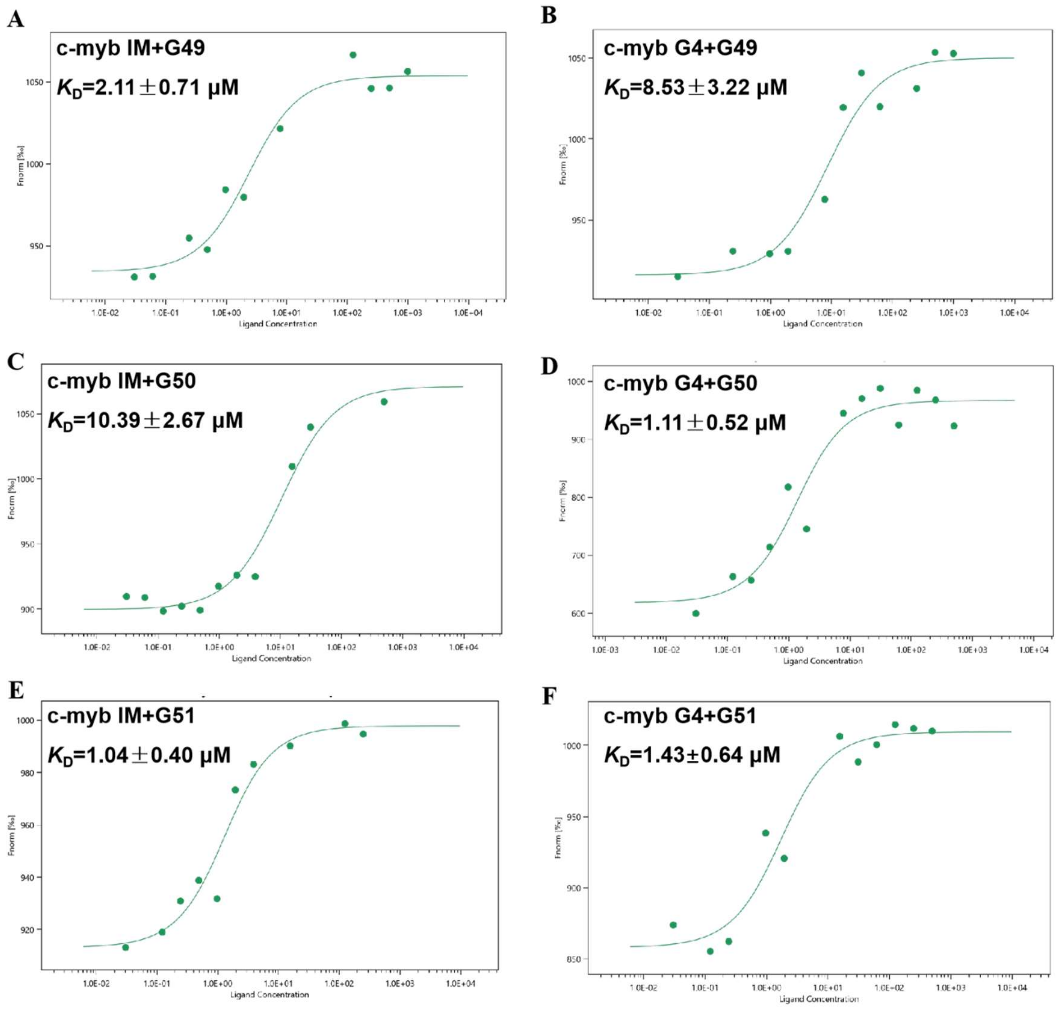

Figure S2. MST experiments were performed for binding of compounds to *c-myb* quadruplex

structures. The binding constants K_D values of compounds **G49**, **G50**, and **G51** with *c-myb* IM were determined to be 2.11 ± 0.71 μM, 10.39 ± 2.67 μM, and 1.04 ± 0.40 μM, respectively in MES buffer at pH 5.5 (A, C, E). The binding constant K_D values of compounds **G49**, **G50**, and **G51** with *c-myb* G4 were determined to be 8.53 ± 3.22 μM, 1.11 ± 0.52 μM, and 1.43 ± 0.64 μM, respectively in Tris-HCl buffer at pH 7.4 (B, D, F).

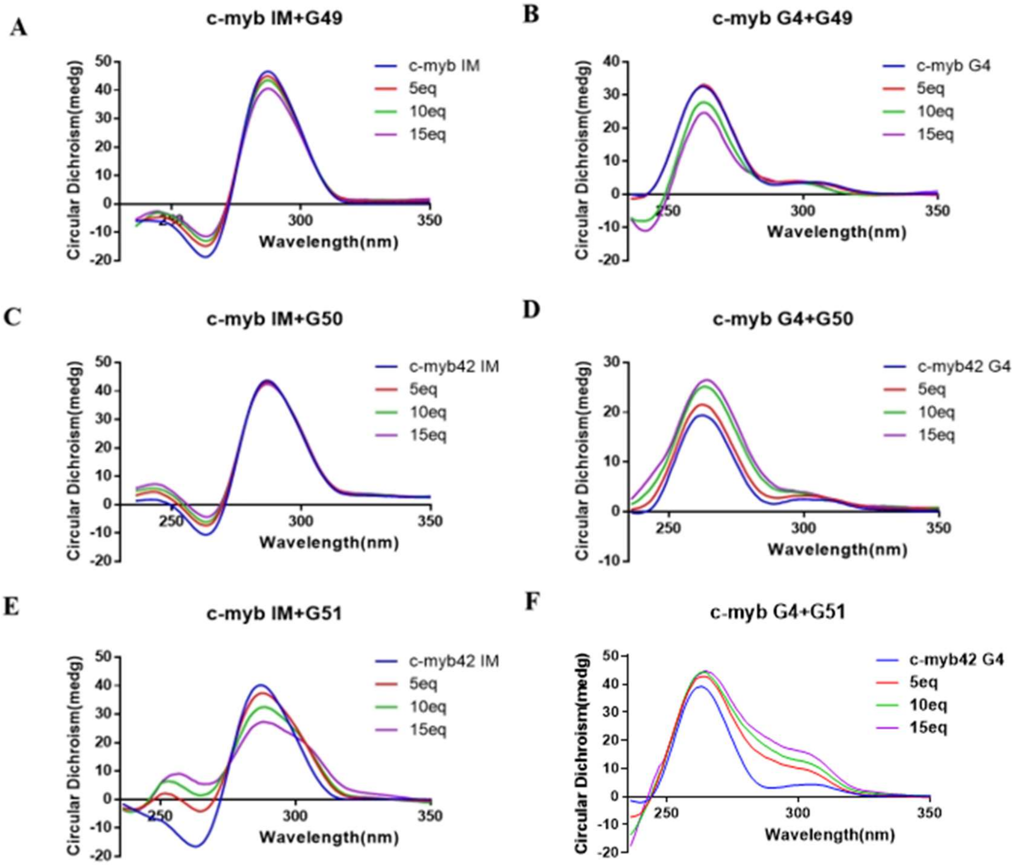

Figure S3. Effects of **G49**, **G50**, **G51** on *c-myb* promoter quadruplex structures were studied by using CD. (A) CD spectrum of *c-myb* IM structure with different equivalent **G49** in BPES buffer at pH 5.5. (B) CD spectrum of *c-myb* G4 structure with different equivalent **G49** in Tris-HCl buffer at pH 7.4. (C) CD spectrum of *c-myb* IM structure with different equivalent **G50** in BPES buffer at pH 5.5. (D) CD spectrum of *c-myb* G4 structure with different equivalent **G50** in Tris-HCl buffer at pH 7.4. (E) CD spectrum of *c-myb* IM structure with different equivalent **G51** in BPES buffer at pH 5.5. (F) CD spectrum of *c-myb* G4 structure with different equivalent **G51** in Tris-HCl buffer at pH 7.4.

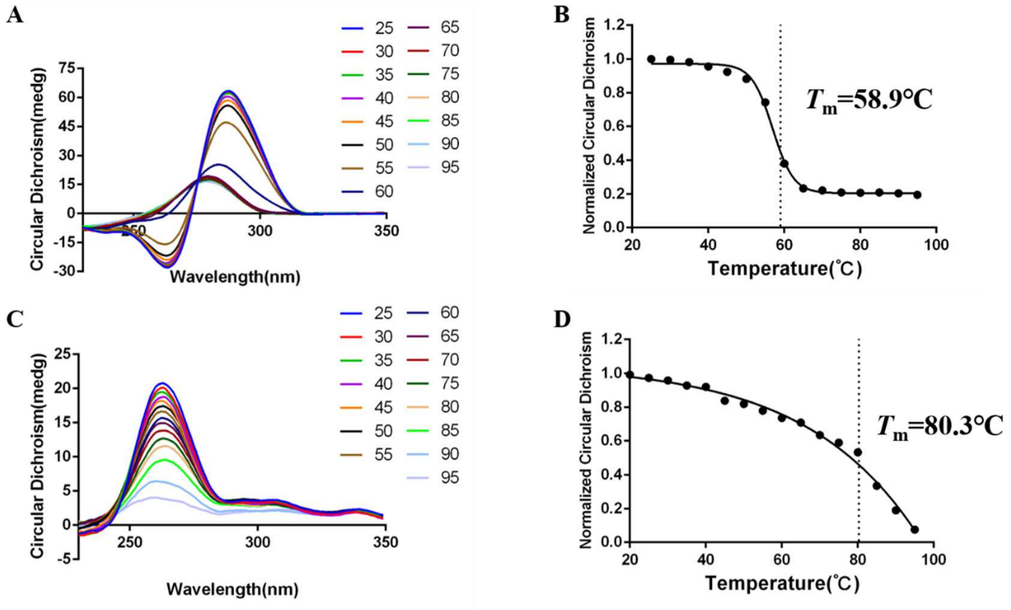

Figure S4. The thermodynamic stabilities of *c-myb* i-motif and G4 structures were measured using CD melting spectra. (A) CD melting spectra of *c-myb* IM were taken at temperatures ranged from 20 °C to 95 °C in BPES buffer at pH 5.5. (B) T_m value was determined to be 58.9 °C through nonlinear curve fitting. (C) CD melting spectra of *c-myb* G4 were taken at temperatures ranged from 20 °C to 95 °C in Tris-HCl buffer at pH 7.4. (D) T_m value was determined to be 80.3 °C through nonlinear curve fitting.

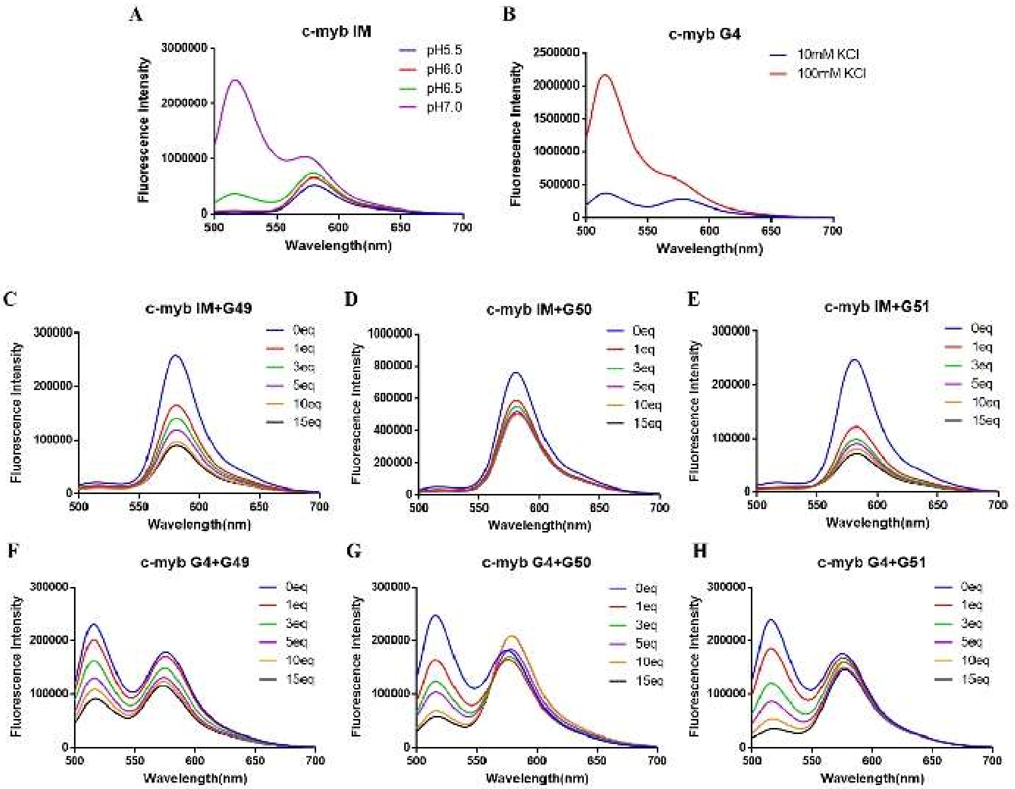

Figure S5. Fluorescence response experiments were carried out to study the effects of compounds on the formation of *c-myb* promoter quadruplex structures. (A) Fluorescence response curves of double-labeled *c-myb* IM oligomer annealed with BPES buffer at different pH. (B) Fluorescence response curves of *c-myb* G4 oligomer annealed with pH 7.4 Tris-HCl buffer at different K^+ concentration. (C) The fluorescence response curves were measured with double-labeled *c-myb* IM oligomer with increasing concentration of **G49**. (D) The fluorescence response curves were measured with double-labeled *c-myb* IM oligomer with increasing concentration of **G50**. (E) The fluorescence response curves were measured with double-labeled *c-myb* IM oligomer with increasing concentration of **G51**. (F) The fluorescence response curves were measured with double-labeled *c-myb* G4 oligomer with increasing concentration of **G49**. (G) The fluorescence response curves were measured with double-labeled *c-myb* G4 oligomer with increasing concentration of **G50**. (H) The fluorescence response curves were measured with double-labeled *c-myb* G4 oligomer with increasing concentration of **G51**.

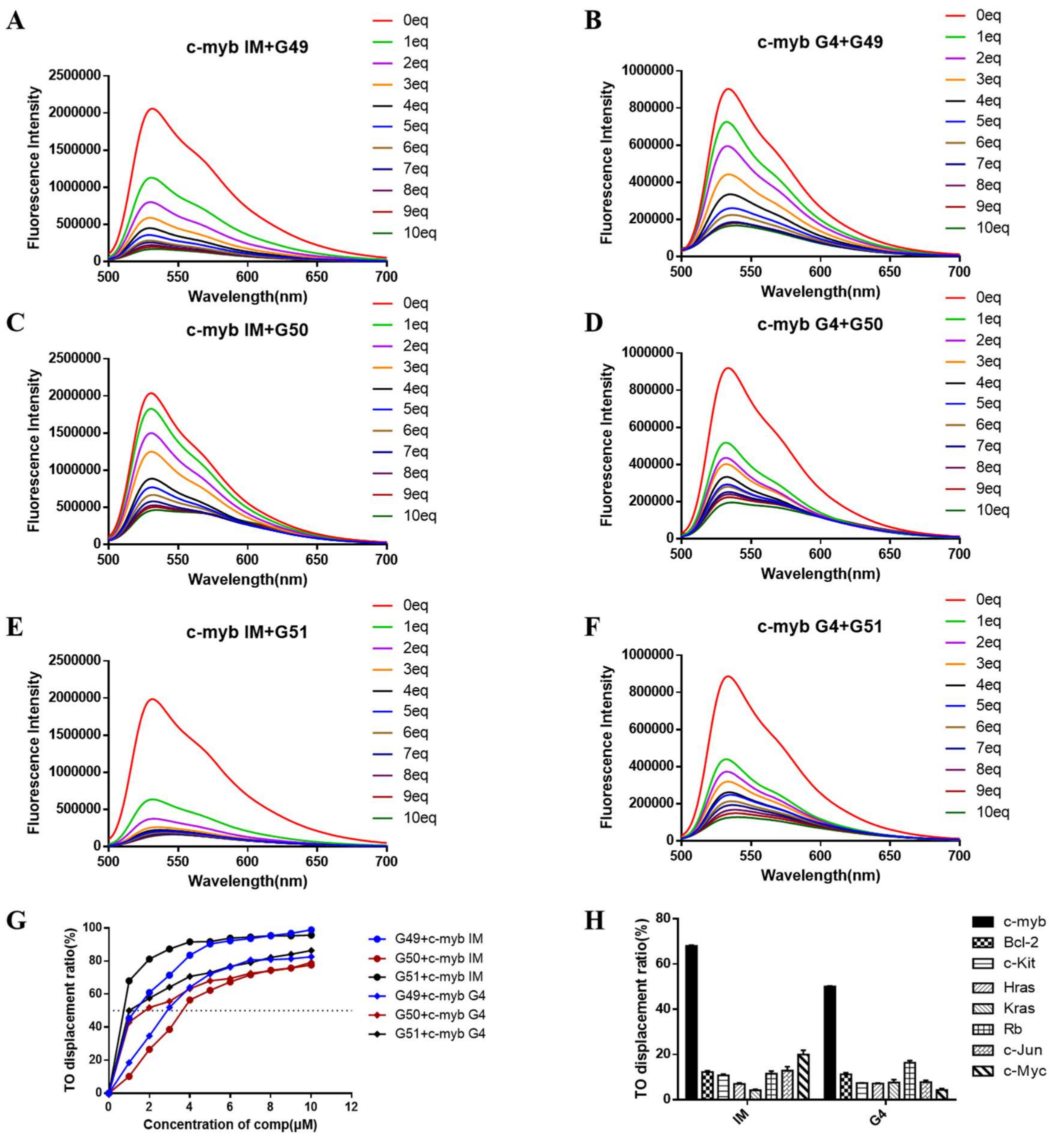

Figure S6. TO displacement experiment for quadruplexes. (A-B) Fluorescence spectra of TO displacement experiment for *c-myb* IM and G4 with increasing concentration of **G49**. (C-D) Fluorescence spectra of TO displacement experiment for *c-myb* IM and G4 with increasing concentration of **G50**. (E-F) Fluorescence spectra of TO displacement experiment for *c-myb* IM and G4 with increasing concentration of **G51**. (G) The plots of displacement ratio for *c-myb* IM/G4 against concentration of **G49**, **G50**, and **G51**. (H) Histogram of TO displacement ratio of 1 eq **G51** for different gene promoter quadruplexes with λ_{ex} at 480 nm and λ_{em} at 530 nm. All experiments were repeated for three times in parallel. The data were expressed as mean ± SEM.

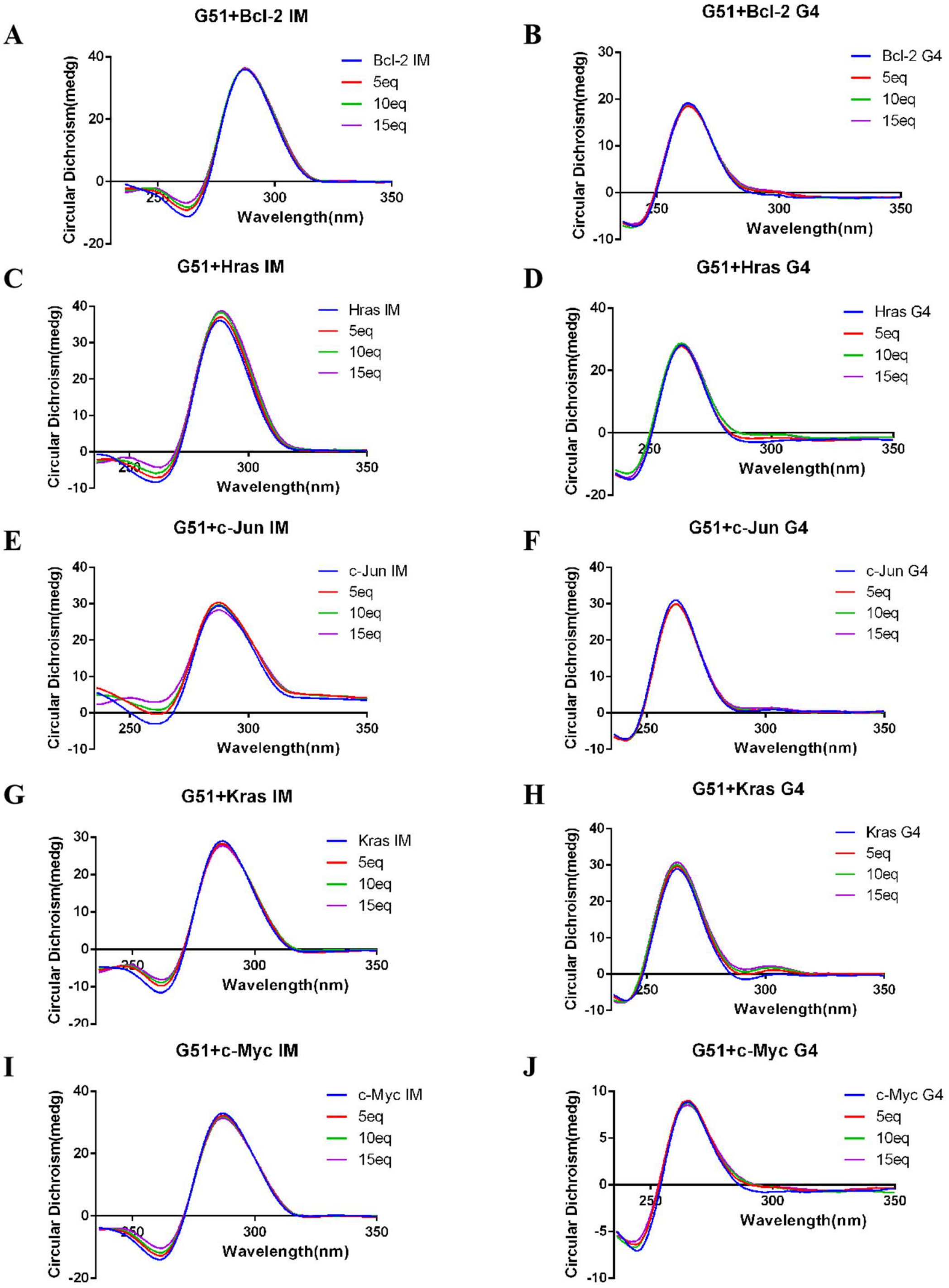

Figure S7. CD experiment was performed for binding of **G51** to other gene quadruplex structures. (A) CD spectrum of *Bcl-2* IM structure with different equivalent **G51** in BPES buffer at pH 5.5. (B) CD spectrum of *Bcl-2* G4 structure with different equivalent **G51** in Tris-HCl buffer at pH 7.4. (C) CD spectrum of *Hras* IM structure with different equivalent **G51** in BPES buffer at pH 5.5. (D) CD spectrum of *Hras* G4 structure with different equivalent **G51** in Tris-HCl buffer

at pH 7.4. (E) CD spectrum of *c-Jun* IM structure with different equivalent **G51** in BPES buffer at pH 5.5. (F) CD spectrum of *c-Jun* G4 structure with different equivalent **G51** in Tris-HCl buffer at pH 7.4. (G) CD spectrum of *Kras* IM structure with different equivalent **G51** in BPES buffer at pH 5.5. (H) CD spectrum of *Kras* G4 structure with different equivalent **G51** in Tris-HCl buffer at pH 7.4. (I) CD spectrum of *c-myc* IM structure with different equivalent **G51** in BPES buffer at pH 5.5. (J) CD spectrum of *c-myc* G4 structure with different equivalent **G51** in Tris-HCl buffer at pH 7.4.

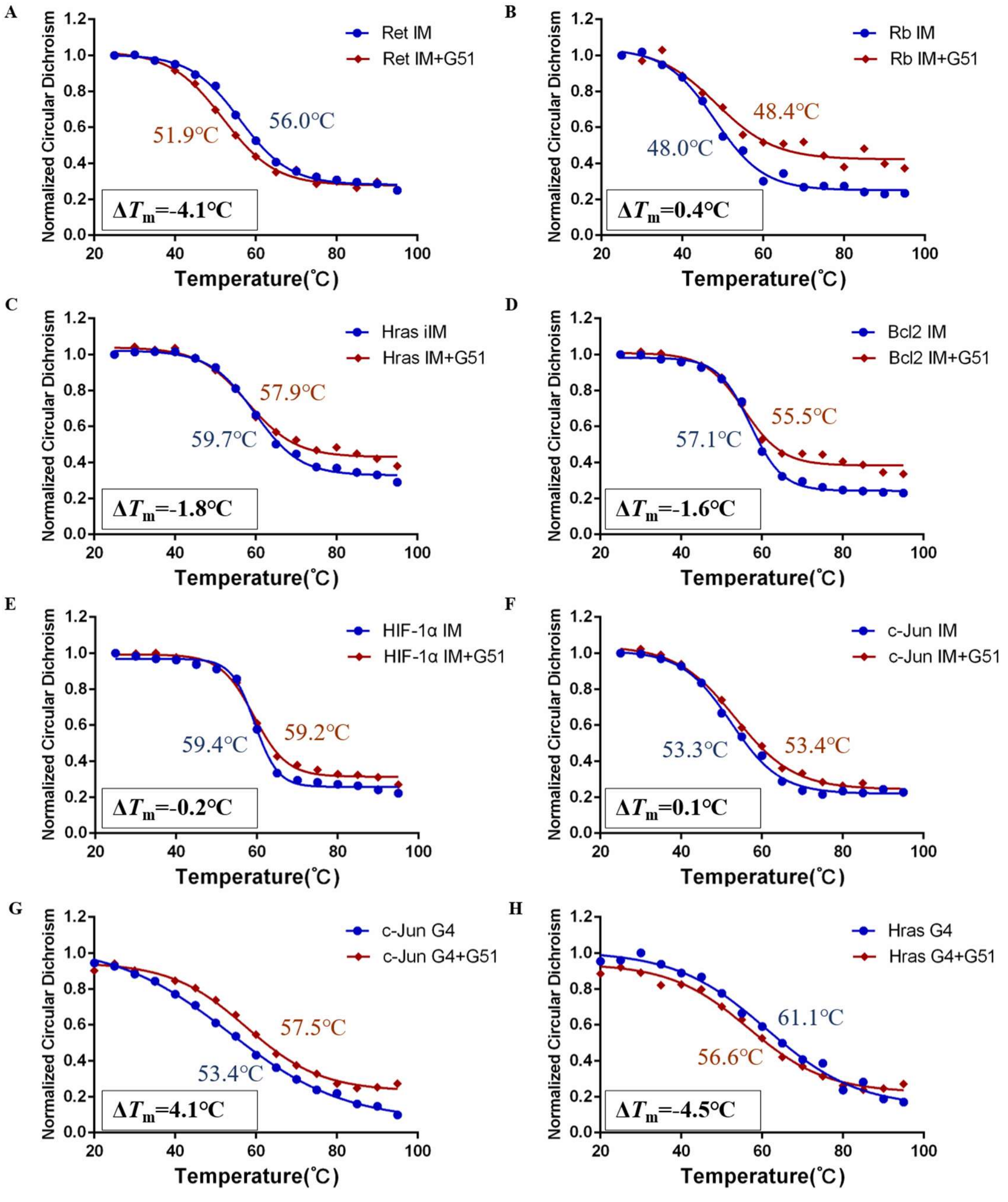

Figure S8. CD melting experiments were carried out to study the effect of **G51** on stabilizing

various DNA secondary structures. (A) *Ret* i-motif; (B) *Rb* i-motif; (C) *Hras* i-motif; (D) *Bcl-2* i-motif; (E) *HIF-1α* i-motif; (F) *c-Jun* i-motif; (G) *c-Jun* G4; (H) *Hras* G4.

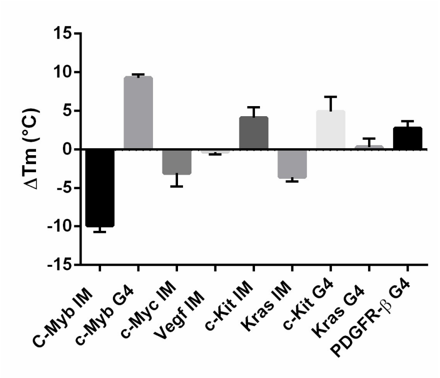

Figure S9. ΔT_m values for various DNA secondary structures interacting with compound **G51** were determined by using FRET melting.

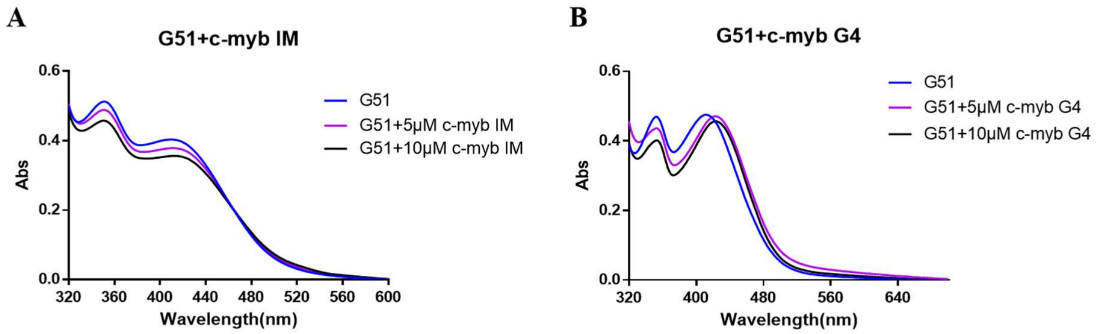

Figure S10. UV titration experiment was performed to study the interaction of **G51** with *c-myb* promoter quadruplex structures. (A) UV-vis spectrum for **G51** with *c-myb* i-motif in BPES buffer at pH 5.5; (B) UV-vis spectrum for **G51** with *c-myb* G4 in Tris-HCl buffer at pH 7.4.

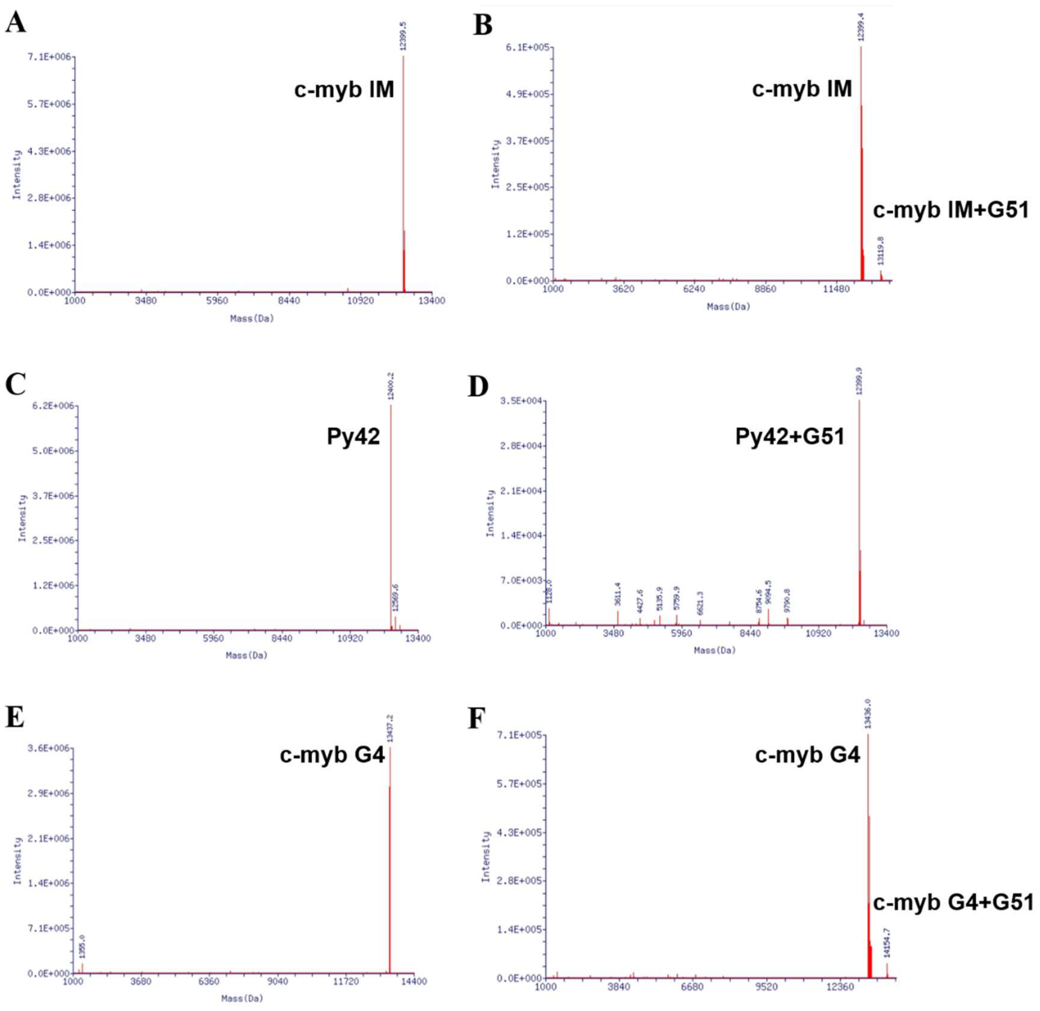

Figure S11. ESI-MS spectra of *c-myb* promoter C-rich (Py42) and G-rich (Pu42) oligomer with or without addition of **G51**. (A) Mass spectrum of Py42 in BPES buffer at pH 5.5. (B) Mass spectrum for binding of **G51** with *c-myb* IM in BPES buffer at pH 5.5. (C) Mass spectrum of Py42 in BPES buffer at pH 7.0. (D) Mass spectrum for binding of **G51** with Py42 in BPES buffer at pH 7.0. (E) Mass spectrum of *c-myb* G4 in BPES buffer at pH 7.4. (F) Mass spectrum for binding of **G51** with *c-myb* G4 in BPES buffer at pH 7.4.

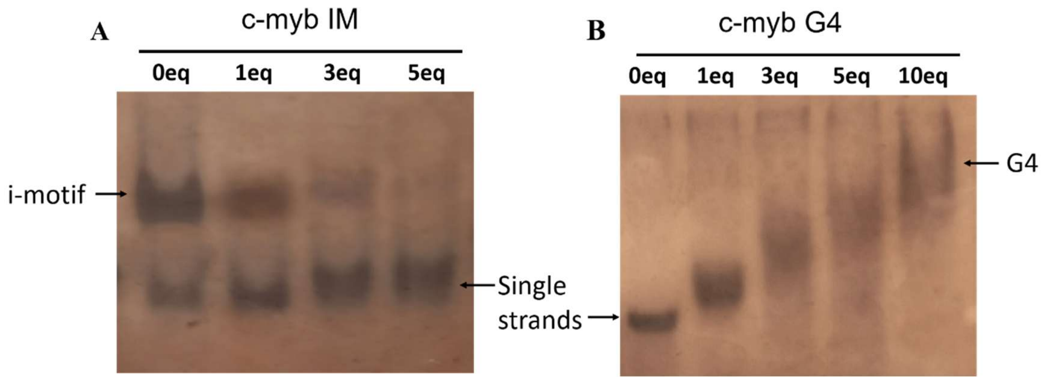

Figure S12. EMSA experiment was performed with silver staining for the effect of **G51** on *c-myb* promoter quadruplex structures. (A) Compound **G51** unfolded *c-myb* i-motif structure in BPES buffer at pH 5.5. Channels from left to right: *c-myb* IM; *c-myb* IM + 1 eq **G51**; *c-myb* IM + 3 eq **G51**; *c-myb* IM + 5 eq **G51**. (B) Compound **G51** induced G-rich oligomers (not annealed) to form G4 structures. Channels from left to right were: *c-myb* G4; *c-myb* G4 + 1 eq **G51**; *c-myb* G4 + 3 eq **G51**; *c-myb* G4 + 5 eq **G51**; *c-myb* G4 + 10 eq **G51**.

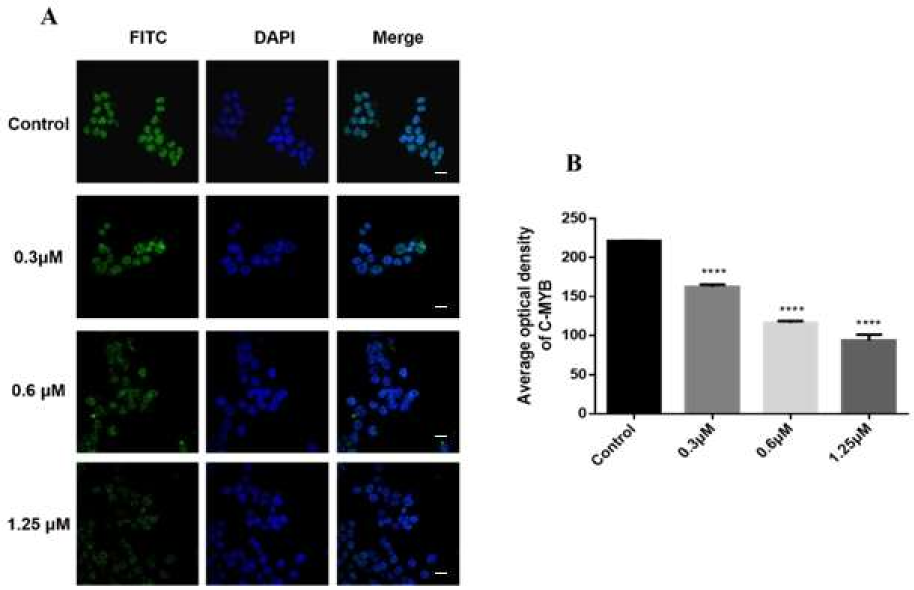

Figure S13. (A) The expression of C-MYB protein in HCT116 cells incubated with **G51** for 48h was analyzed by using immunofluorescence (Scale bars: 20 μm). (B) Experiments were repeated three times and data were showed as mean ± SEM. (****) $P < 0.0001$.

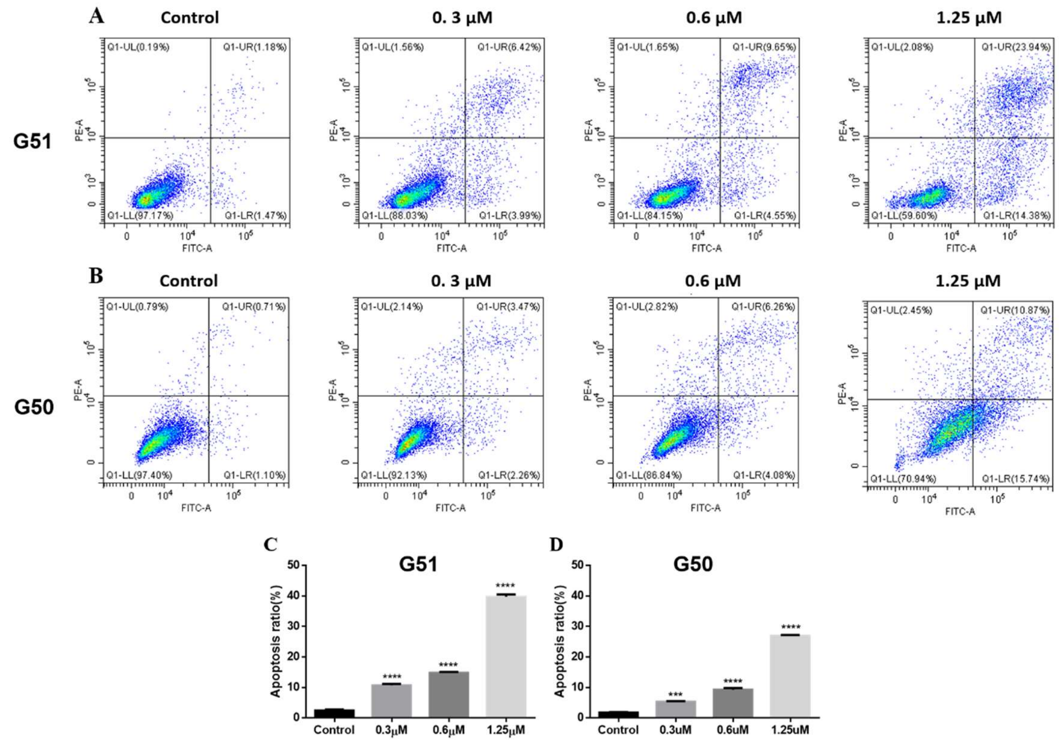

Figure S14. The effects of **G51** and **G50** on apoptosis of HCT116 cells. (A) Apoptosis of HCT116 cells was determined upon **G51** treatment, which was analyzed as shown in (C). (B) Apoptosis of HCT116 cells was determined upon **G50** treatment, which was analyzed as shown in (D). Experiments were repeated three times and data were showed as mean ± SEM. (***) P < 0.001, and (****) P < 0.0001.

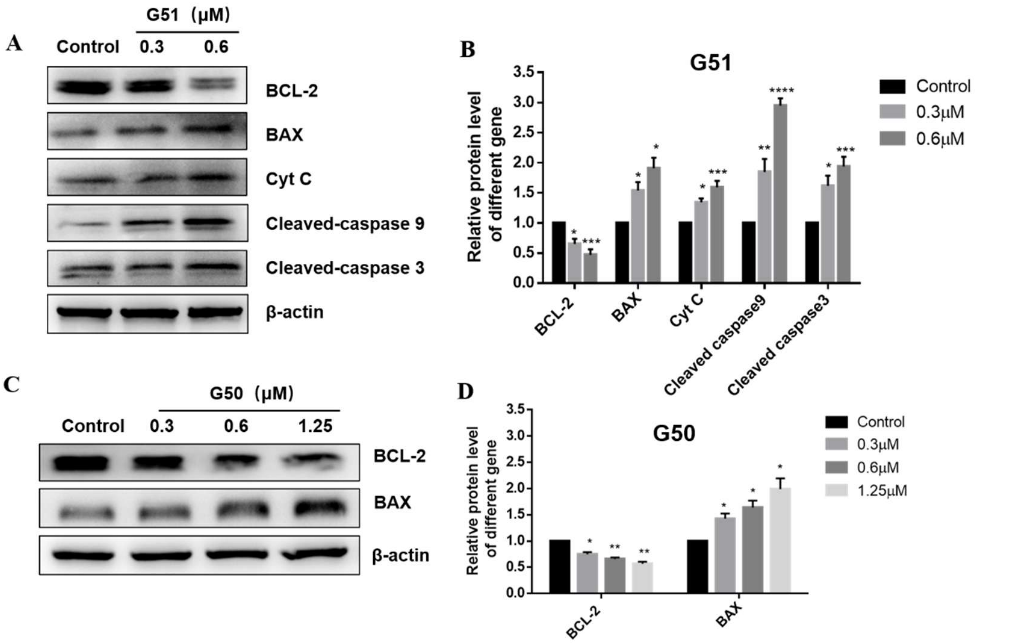

Figure S15. Effects of compounds **G51** and **G50** on the expressions of apoptosis-related proteins in HCT116 cells. (A) The expressions of BCL-2, BAX, Cyt C, Cleaved caspase3, Cleaved caspase9 proteins in HCT116 cells after **G51** treatment were analyzed, with quantitative results as shown in (B). (C) The expressions of BCL-2, BAX proteins in HCT116 cells after **G50** treatment were analyzed, with quantitative results as shown in (D). Experiments were repeated three times and data were showed as mean ± SEM. (*) P < 0.05, (**) P < 0.01, (***) P < 0.001, and (****) P < 0.0001.

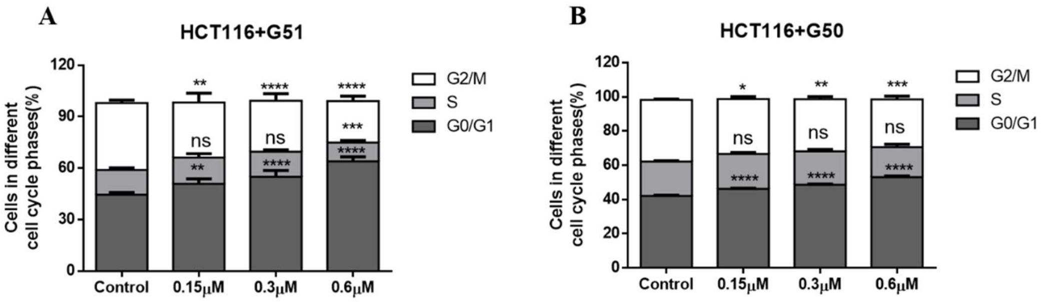

Figure S16. Cell cycle analysis of HCT116 cells upon treatment with compounds **G51** and **G50** for 24h. (A) Statistical graph for the number of cells in each cycle after treatment with **G51**. (B) Statistical graph for the number of cells in each cycle after treatment with **G50**. Experiments were repeated three times and data were showed as mean ± SEM. (ns) not significant, (*) P < 0.05, (**) P < 0.01, (***) P < 0.001, and (****) P < 0.0001.

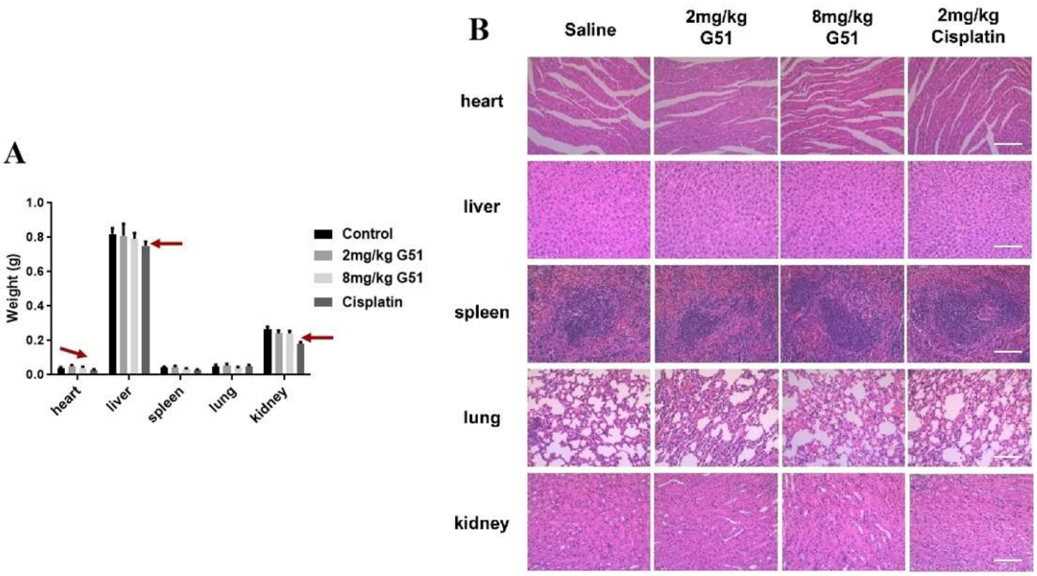

Figure S17. Comparison of vital organs for mice in different treatment groups. (A) The relative

proportion of organs to body weight. (B) HE staining images of various organs (Scale bars: 200 μm).

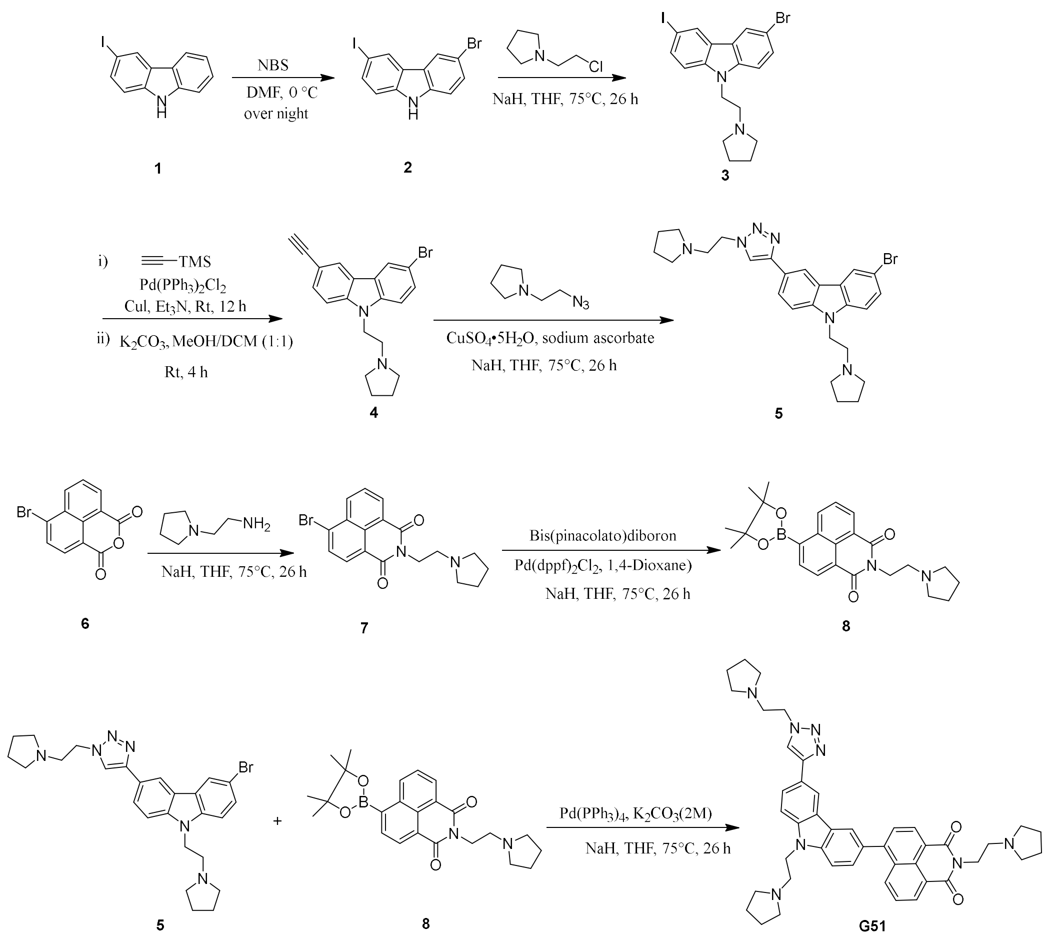

Scheme SI. Synthetic pathway for carbazole derivative **G51**.

Table S1. Oligonucleotides used in this study

Oligomer	Sequence
c-myb IM	5'-TCCTCCTCCTCCTTCTCCTCCTCCTCCGTGACCTCCTCC TCC -3'
c-myb G4	5'-GGAGGAGGAGGTCACGGAGGAGGAGGAGAAGGAGGA GGAGGA -3'
Bcl-2 IM	5'-CAGCCCCGCTCCCGCCCCCTTCCTCCCGCGCCCGCCCCT 3'

Bcl-2 G4	5’-GGGCGGGCGCGGGAGGAAGGGGGCGGG -3’
c-Jun IM	5’-TAACCCCCTCCCCCTCCCCCCTTTAAT -3’
c-Jun G4	5’-AAGGGGGGAGGGGGAGGGGGTT -3’
Kras IM	5’-CCTCCCCCTCTTCCCTCTTCCCACACCGCCCT-3’
Kras G4	5’-AGGGCGGTGTGGGAAGAGGGAAGAGGGGGAGG -3’
Hras IM	5’-ACCGCGCGCCCCCGCCCCCGCCCCGCCCCGGCCTCG -3’
Hras G4	5’-TTGAGGGTGGGTAGGGTGGGTAAA-3’
Ret IM	5’-CCCCGCCCCGCCCCGCCCCTA-3’
Rb IM	5’-GCCGCCCAAAACCCCCCG -3’
HIF-1α IM	5’-GCCCGAGCGCGCCTCCGCCCTTGCCCGCCCCCTG -3’
F-*c-myb* IM-T	5′-FAM-TCCTCCTCCTCCTTCTCCTCCTCCTCCGTGACCTCC TCCTCC-TAMRA-3′
F-*c-myb* G4-T	5′-FAM-GGAGGAGGAGGTCACGGAGGAGGAGGAGAAGGA GGAGGAGGA-TAMRA-3′
5’-biotin *c-myb* IM	5′-Biotin-TCCTCCTCCTCCTTCTCCTCCTCCTCCGTGACCTCC TCCTCC-3’
5’-biotin *c-myb* G4	5′-Biotin-GGAGGAGGAGGTCACGGAGGAGGAGGAGAAGGA GGAGGAGGA -3’
5’-biotin *c-myb* G4	5′-Biotin-GGAGGAGGAGGTCACGGAGGAGGAGGAGAAGGA GGAGGAGGA -3’

Table S2. Equilibrium binding constants (K_D) determined by using SPR

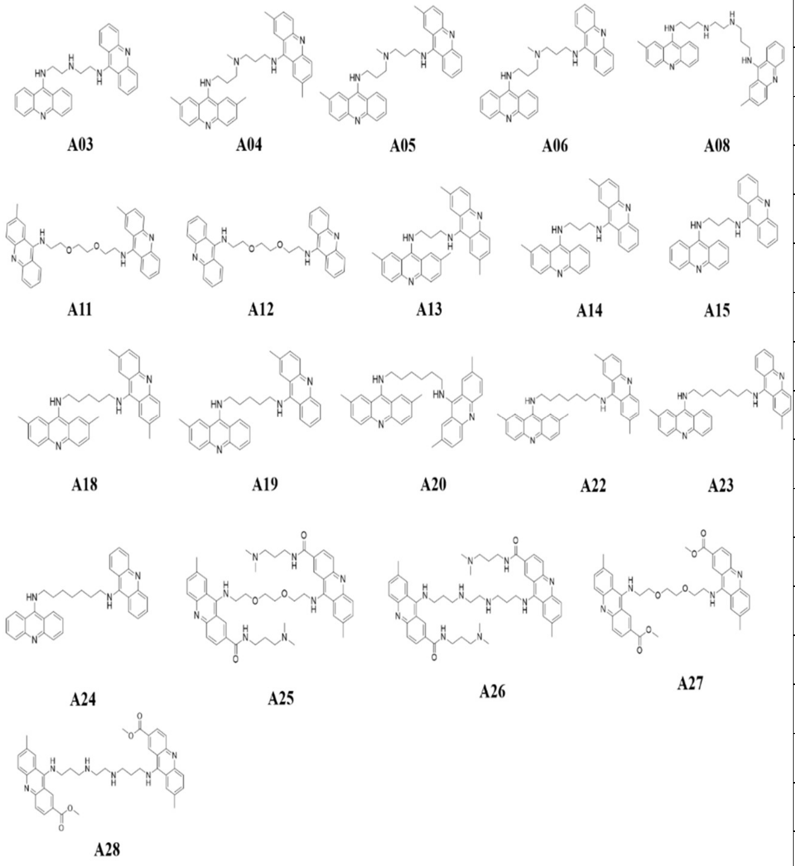

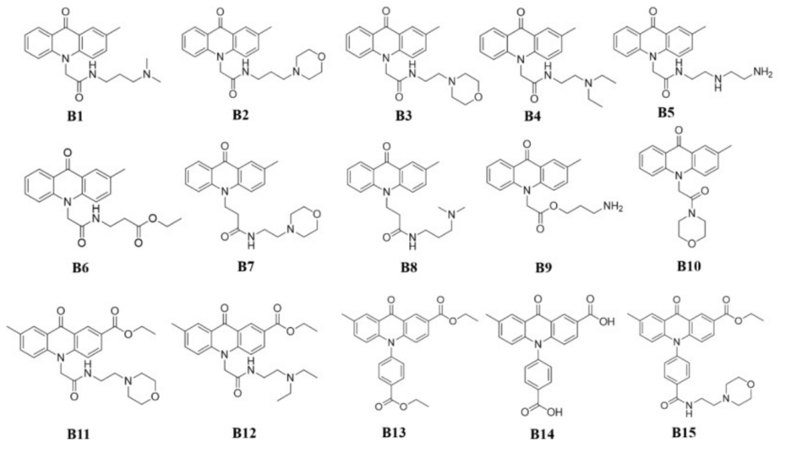

compound	K_D (RB promoter i-motif)
**A03**	**19.90**
**A04**	**33.90**
**A05**	>50
**A06**	**7.58**
**A08**	**5.60**
**A11**	>50
**A12**	>50
**A13**	>50
**A14**	>50
**A15**	>50
**A18**	>50
**A19**	>50
**A20**	>50
**A22**	>50
**A23**	>50
**A24**	**22.50**
**A25**	>50
**A26**	>50
**A27**	>50
**A28**	>50
**B1**	>50
**B2**	>50
**B3**	>50
**B4**	>50
**B5**	>50
**B6**	>50
**B7**	>50
**B8**	>50
**B9**	>50
**B13**	>50
**B14**	>50

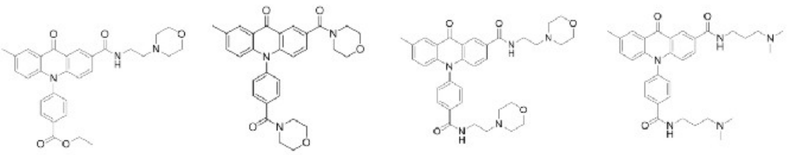 B16 B17 B18 B19 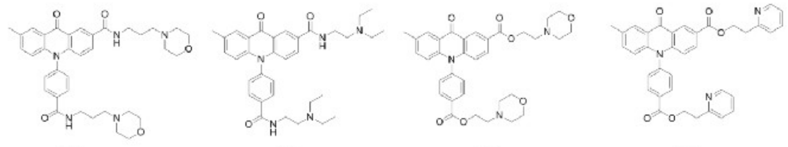 B20 B21 B22 B23	**B15**	>50
	**B16**	>50
	**B17**	>50
	**B18**	>50
	**B19**	>50
	**B20**	>50
	**B21**	>50
	**B22**	>50
	**B23**	>50
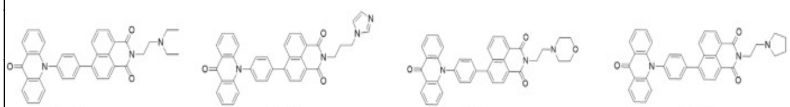 C69 C70 C71 C72 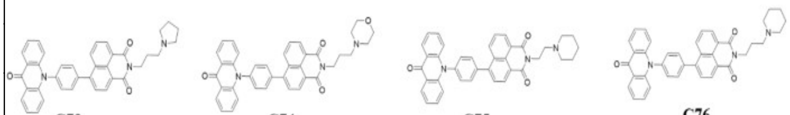 C73 C74 C75 C76 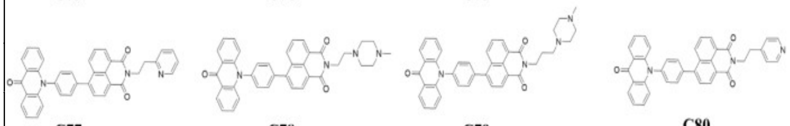 C77 C78 C79 C80 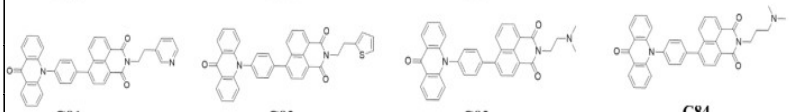 C81 C82 C83 C84 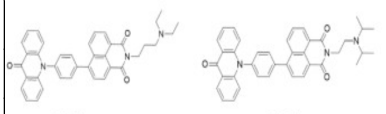 C85 C86	**C69**	>50
	**C70**	>50
	**C71**	>50
	**C72**	>50
	**C73**	>50
	**C74**	>50
	**C75**	>50
	**C76**	>50
	**C77**	>50
	**C78**	>50
	**C79**	>50
	**C80**	>50
	**C81**	>50
	**C82**	>50
	**C83**	>50
	**C84**	>50
	**C85**	>50
	**C86**	>50
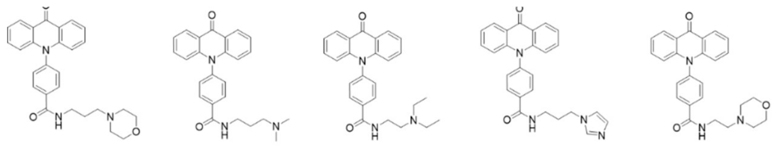 E40 E41 E42 E43 E44 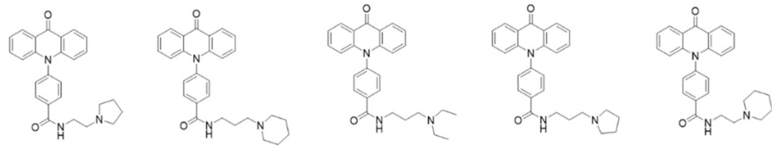 E45 E46 E47 E48 E49	**E40**	>50
	**E41**	>50
	**E42**	>50
	**E43**	>50
	**E44**	>50
	**E45**	>50
	**E46**	>50

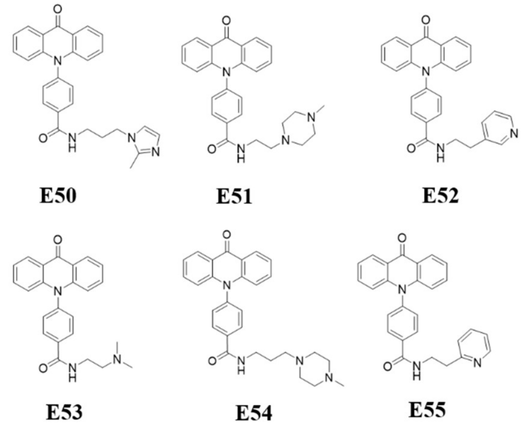	**E47**	>50
	**E48**	>50
	**E49**	>50
	**E50**	>50
	**E51**	>50
	**E52**	>50
	**E53**	>50
	**E54**	>50
	**E55**	>50
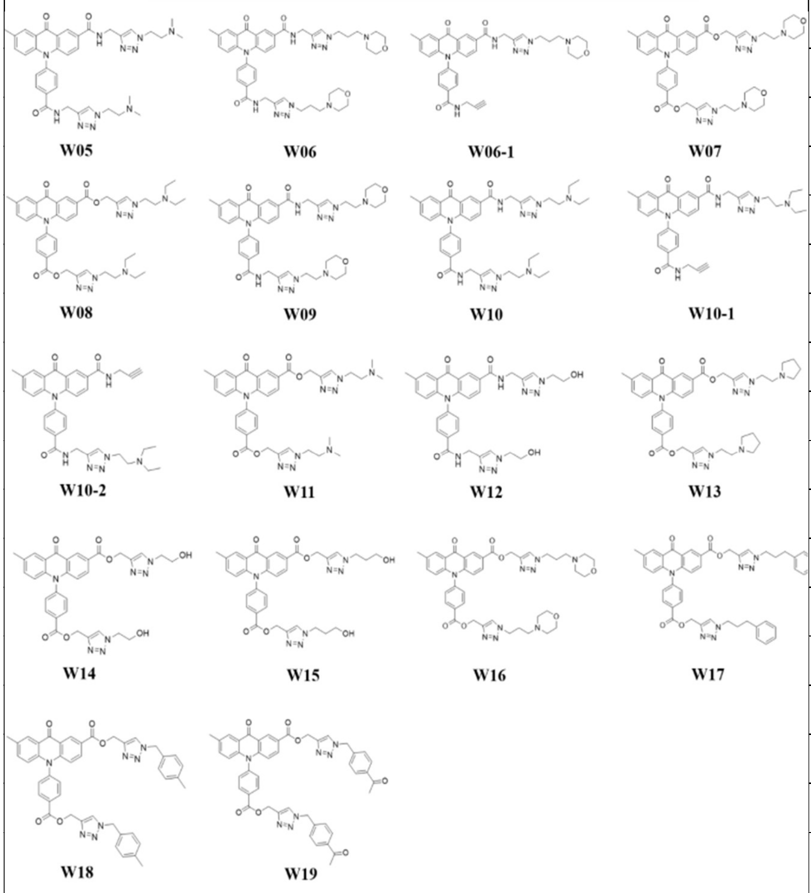	**W05**	>50
	**W06**	>50
	**W06-1**	>50
	**W07**	>50
	**W08**	>50
	**W09**	>50
	**W10**	>50
	**W10-1**	>50
	**W10-2**	>50
	**W11**	>50
	**W12**	>50
	**W13**	>50
	**W14**	>50
	**W15**	>50
	**W16**	>50
	**W17**	>50
	**W18**	>50
	**W19**	>50
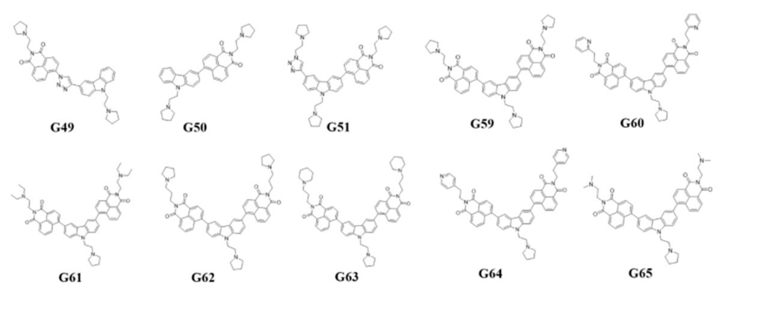	**G49**	**3.34**
	**G50**	**12.90**
	**G51**	**0.58**
	**G59**	>50
	**G60**	>50
	**G61**	>50
	**G62**	>50

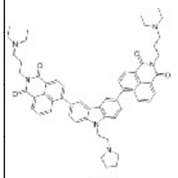 G66 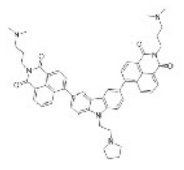 G67 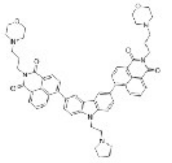 G68 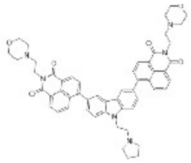 G69	**G63**	>50
	**G64**	>50
	**G65**	>50
	**G66**	>50
	**G67**	>50
	**G68**	>50
	**G69**	>50
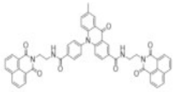 N1 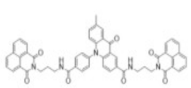 N2 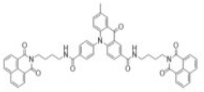 N3 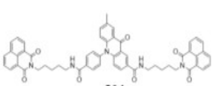 N4 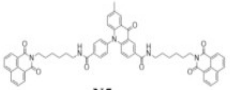 N5 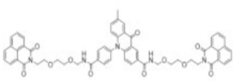 N6 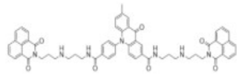 N7 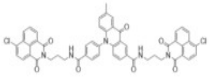 N8 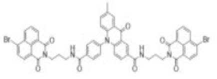 N9 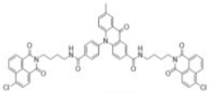 N10 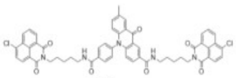 N11 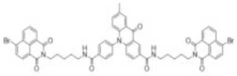 N12 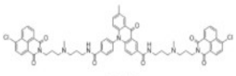 N13 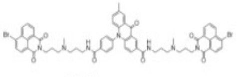 N14	**N1**	>50
	**N2**	>50
	**N3**	>50
	**N4**	>50
	**N5**	>50
	**N6**	>50
	**N7**	>50
	**N8**	>50
	**N9**	>50
	**N10**	>50
	**N11**	>50
	**N12**	>50
	**N13**	>50
	**N14**	>50
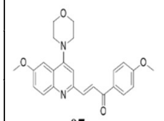 07 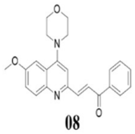 08 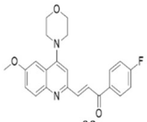 09 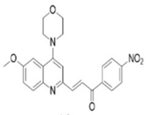 10 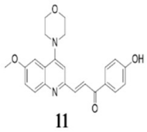 11 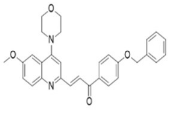 12 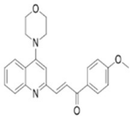 13 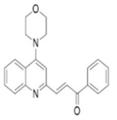 14 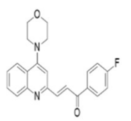 15 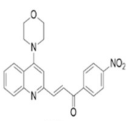 16 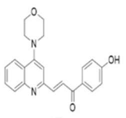 17 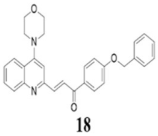 18 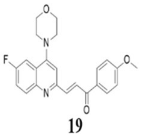 19 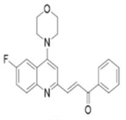 20 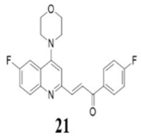 21	**07**	>50
	**08**	>50
	**09**	>50
	**10**	>50
	**11**	>50
	**12**	>50
	**13**	>50
	**14**	>50
	**15**	>50
	**16**	>50
	**17**	>50
	**18**	>50
	**19**	>50
	**20**	>50

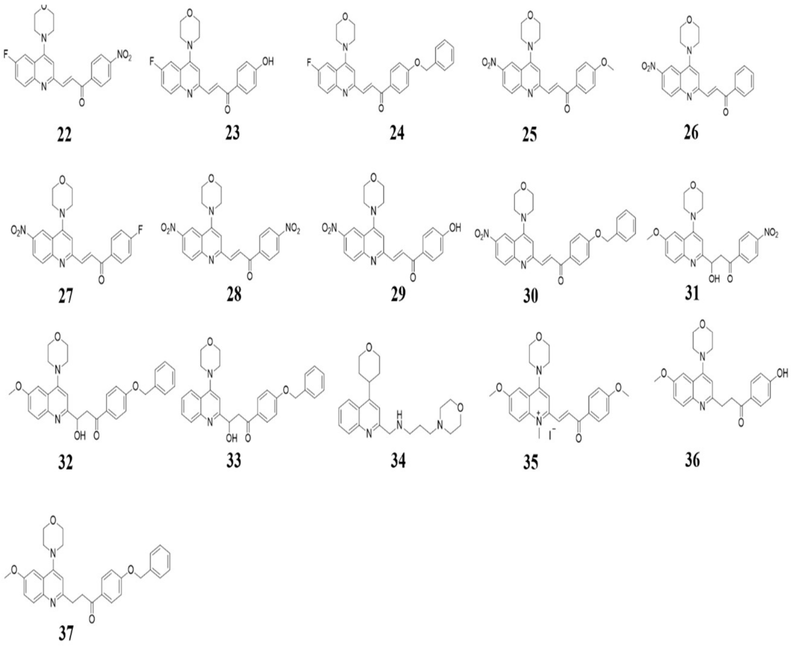	**21**	>50
	**22**	>50
	**23**	>50
	**24**	>50
	**25**	>50
	**26**	>50
	**27**	>50
	**28**	>50
	**29**	>50
	**30**	>50
	**31**	>50
	**32**	>50
	**33**	>50
	**34**	>50
	**35**	>50
	**36**	>50
	**37**	>50

Table S3. Binding affinity of **G51** to different i-motifs and G4s determined by using SPR

Sequence	K_D (μM)	
	**IM**	**G4**
c-myb	0.58	1.10
Kirs	13.40	27.40
Braf	13.80	32.30
ssRET	18.90	- [a]
c-Jun	18.70	32.90
Rb	7.70	14.50
c-myc	10.30	37.30
Bcl-2	18.50	23.60
c-Kit	25.20	- [a]
Telc	26.20	- [a]

Ret	7.71	21.50
Kras	- [a]	27.50
HIF-1α	- [a]	16.70
Hras	- [a]	29.10

[a] : not determined

Table S4. Binding affinity of **G51** to different i-motifs and G4s determined by using MST

**Sequence**	**K_D (μM)**	
	**IM**	**G4**
c-myb	1.04	1.43
HIF-1α	14.19	- [a]
c-Kit	33.17	- [a]
Kras	20.59	19.90
VEGF	30.76	12.77
c-Jun	34.42	[a] >50
Ret	18.00	- [a]
Rb	20.93	10.47

[a] : not determined

Table S5. IC_{50} (μM) values were determined for effects of **G49**, **G50** and **G51** on various types of cells by using MTT assay

**Comp.**	**IC_{50} (μM)**								
	**HCT116**	**SW620**	**RKO**	**SW480**	**DLD1**	**A549**	**HGC-27**	**Hela**	**NCM460**
**G49**	**3.3 ± 0.3**	**4.0 ± 0.7**	**4.9 ± 0.4**	- [a]	**-** [a]	**6.9 ± 1.1**	**12.6 ± 2.6**	**9.9 ± 2.3**	**-** [a]
**G50**	**4.7 ± 0.8**	**1.1 ± 0.5**	**-** [a]	**4.5 ± 0.6**	**2.8 ± 0.8**	**8.9 ± 2.7**	**16.2 ± 1.8**	**12.5 ± 2.6**	**19.8 ± 0.8**
**G51**	**0.6 ± 0.1**	**0.9 ± 0.8**	**3.9 ± 0.4**	**0.9 ± 0.1**	**1.6 ± 0.5**	**7.2 ± 1.9**	**3.0 ± 0.3**	**8.6 ± 1.5**	**12.8 ± 0.5**

[a] : not determined

Table S6. The oligonucleotides for wild type or deleted *c-myb* promoter used for pGL-3 Basic plasmids, with underlined text indicating quadruplex forming sequence or mutant sequence

pGL-WT *c-myb*	GGTACCGGCGGCGCGAGCGCCGAATGGGAGCGGCGACCCGGCCAGCCC<br>GGCAGCCCCGCGGGCGGCAGCCAGGGCGACCGCGGAGGCGGCGGGCA<br>GGGCGCGTGCGCACTGCAGGGGCGCCAGATTTGGCGGGAGGGGGAGTG<br>TCCAAAGCTCTTTGTTTGATGGCATCTCTGTTTACAGAGTTTACACTTTAATA<br>TCAACCTGTT**TCCTCCTCCTCCTTCTCCTCCTCCTCCGTGACCTCCTCCTC**<br>**C**TCTTTCTCCTGAGAAACTTCGCCCCAGCGGTGCGGAGCGCCGCTGCGC<br>AGCCGGGGAGGGACGCAGGCAGGCGGCGGGCAGCGGGAGGCGGCAGC<br>CCGGTAAGCTT
pGL-Del *c-myb*	GGTACCGGCGGCGCGAGCGCCGAATGGGAGCGGCGACCCGGCCAGCCC<br>GGCAGCCCCGCGGGCGGCAGCCAGGGCGACCGCGGAGGCGGCGGGCA<br>GGGCGCGTGCGCACTGCAGGGGCGCCAGATTTGGCGGGAGGGGGAGTG<br>TCCAAAGCTCTTTGTTTGATGGCATCTCTGTTTACAGAGTTTACACTTTAATA<br>TCAACCTGTTTCTTTCTCCTGAGAAACTTCGCCCCAGCGGTGCGGAGCGC<br>CGCTGCGCAGCCGGGGAGGGACGCAGGCAGGCGGCGGGCAGCGGGAG<br>GCGGCAGCCCGGTAAGCTT
pGL-Mut *c-myb*	GGTACCGGCGGCGCGAGCGCCGAATGGGAGCGGCGACCCGGCCAGCCC<br>GGCAGCCCCGCGGGCGGCAGCCAGGGCGACCGCGGAGGCGGCGGGCA<br>GGGCGCGTGCGCACTGCAGGGGCGCCAGATTTGGCGGGAGGGGGAGTG<br>TCCAAAGCTCTTTGTTTGATGGCATCTCTGTTTACAGAGTTTACACTTTAATA<br>TCAACCTGTT**TCTTCTTCTTCCTTCTCTTCTTCTTCTGTGACTTCTTCTTCC**T<br>CTTTCTCCTGAGAAACTTCGCCCCAGCGGTGCGGAGCGCCGCTGCGCAG<br>CCGGGGAGGGACGCAGGCAGGCGGCGGGCAGCGGGAGGCGGCAGCCC<br>GGTAAGCTT

Table S7. Primers used for qRT-PCR

Primer	Sequence
actin	Forward：GACCTGACTGACTACCTCAT
	reverse：TCGTCATACTCCTGCTTGCT
c-myb	forward：CTATTACCACATTTCTGAAGCACAA

	reverse：TAGCTCATTCTCGGTTGACATTAG
VEGF	forward：GCTACTGCCATCCAATCGAG
	reverse：CTTGGTGAGGTTTGATCCGC
Kras	forward：GTGGAGTATTTGATAGTGTTTAAC
	reverse：TGTATCAAAGAATGGTCCTGCA
c-myc	forward：GGACGACGAGACCTTCATCAA
	reverse：CCAGCTTCTCTGAGACGAGCTT

^{1}H NMR and ^{13}C NMR spectra of carbazole derivatives

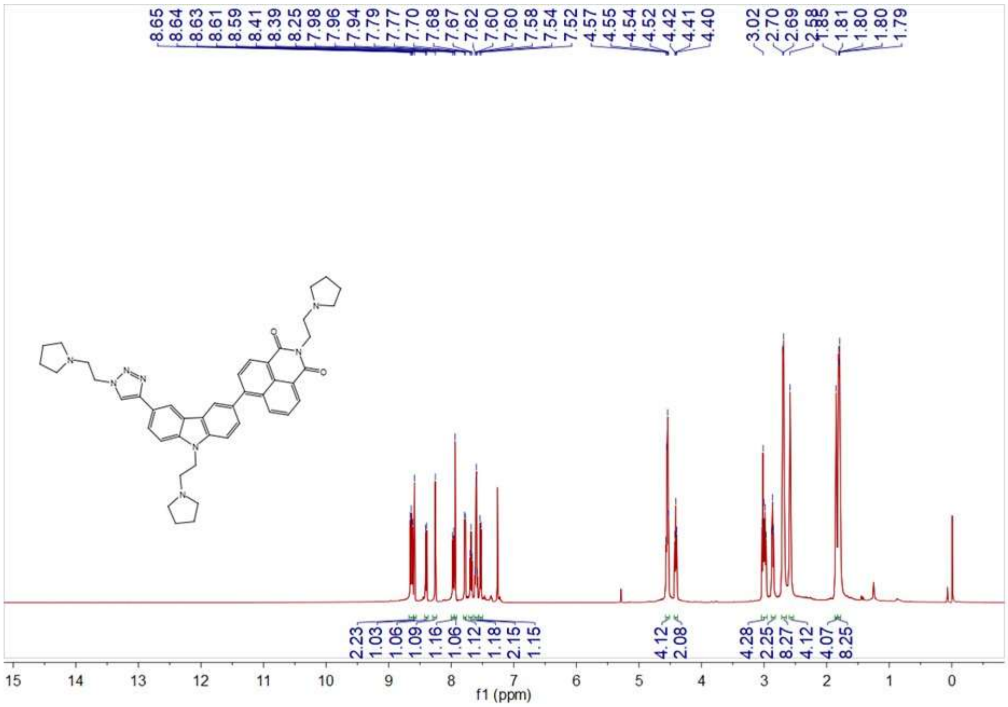

^{1}H NMR spectrum of **G51**

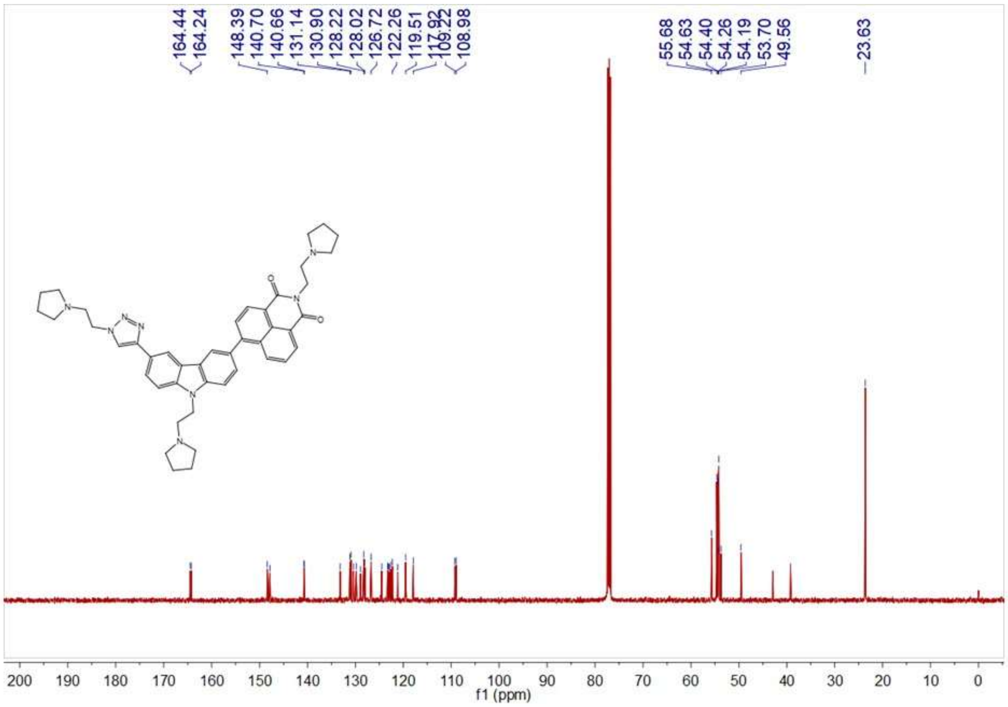

^{13}C NMR spectrum of **G51**

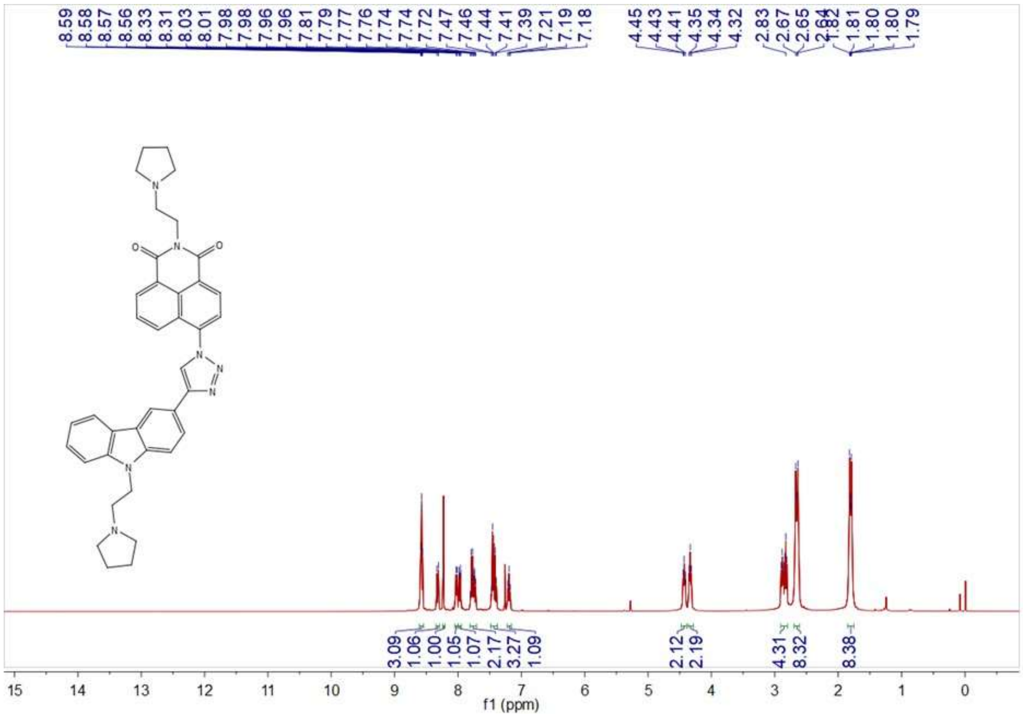

^{1}H NMR spectrum of **G49**

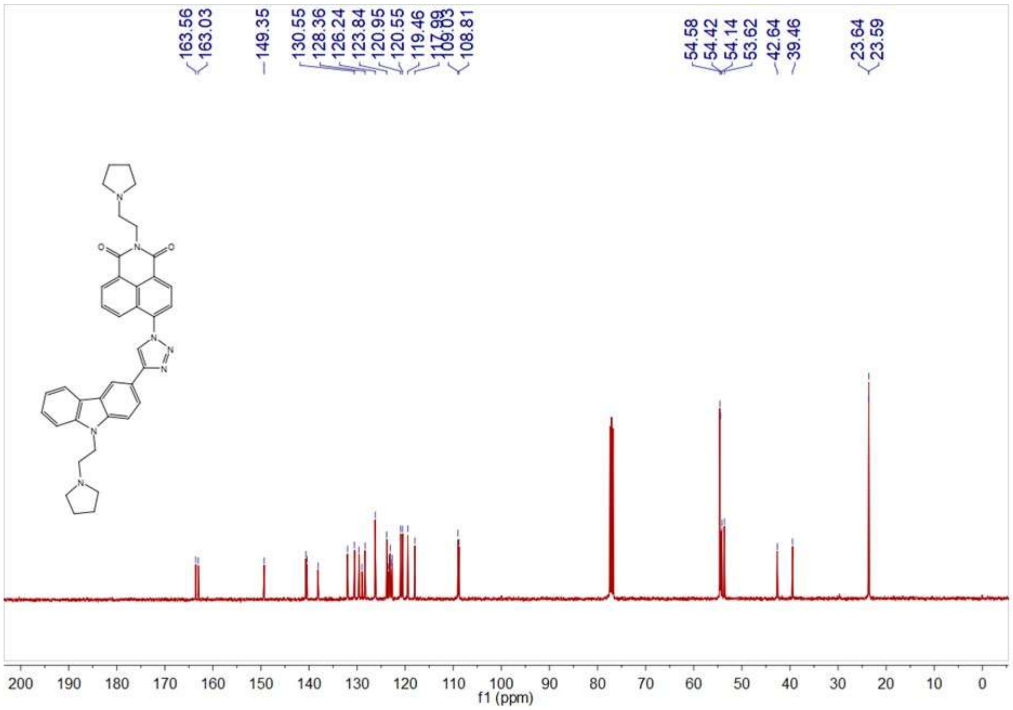

^{13}C NMR spectrum of **G49**

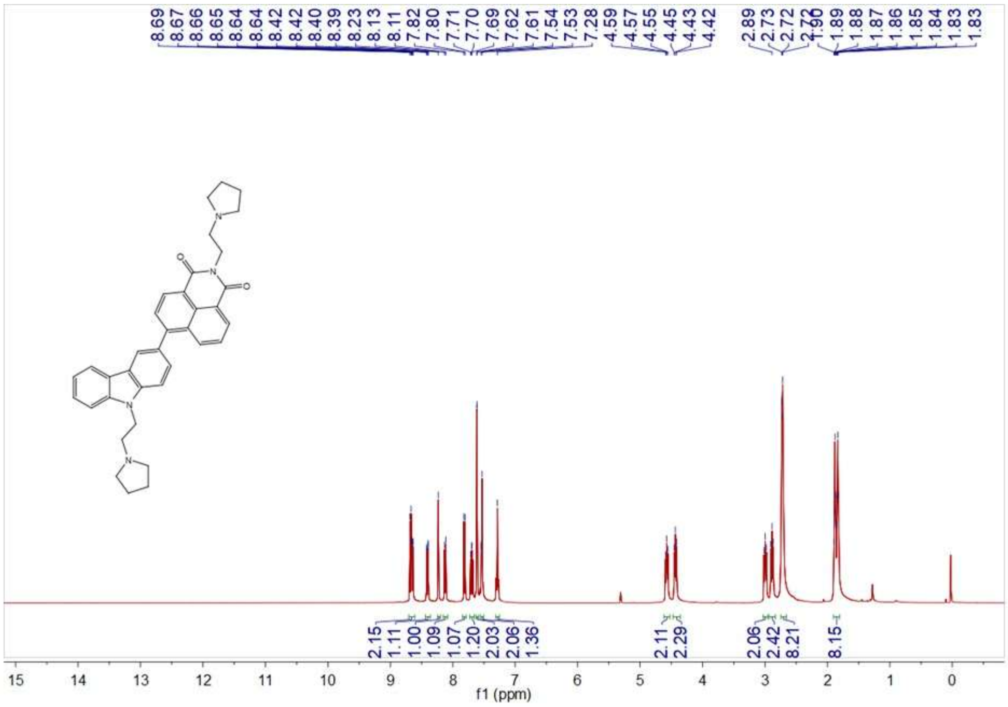

^{1}H NMR spectrum of **G50**

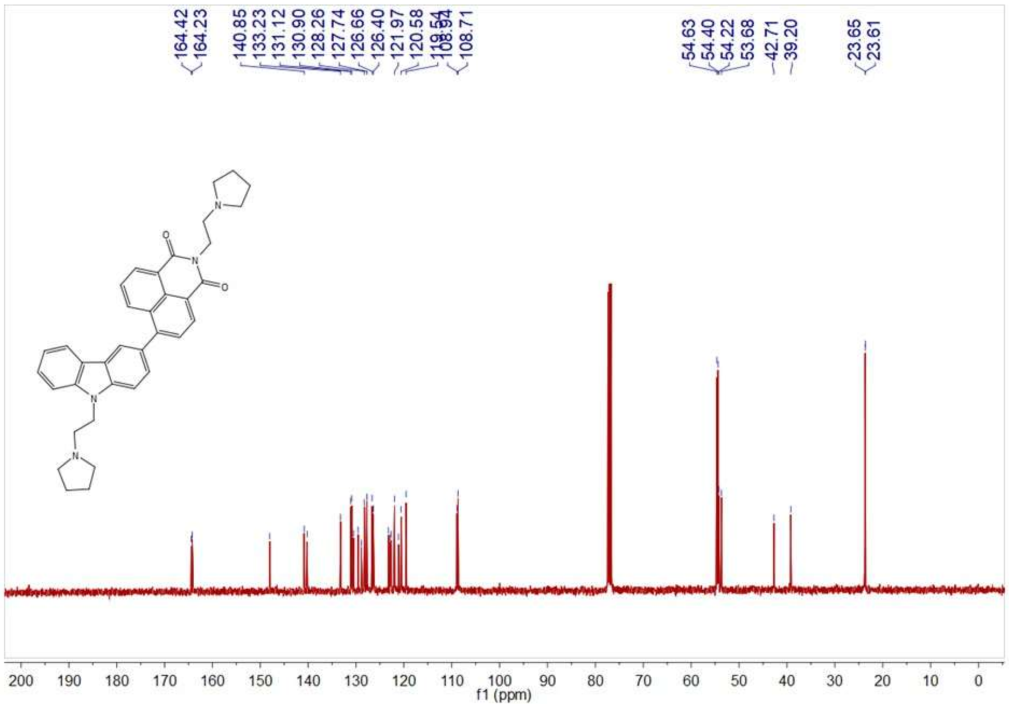

^{13}C NMR spectrum of **G50**
